# Odd–Even Cation Engineering of the Excitation Transport Anisotropy in Two-Dimensional Perovskite Films

**DOI:** 10.1021/acsnano.6c05301

**Published:** 2026-05-20

**Authors:** Jiaxing Du, Marcello Righetto, Maryam Choghaei, Siyu Yan, Christopher A. Wallerius, Klaus Meerholz, Michael B. Johnston, Selina Olthof, Laura M. Herz

**Affiliations:** a Department of Physics, 6396 University of Oxford, Clarendon Laboratory, Parks Road, Oxford OX1 3PU, United Kingdom; b Department of Chemical Science, Università degli Studi di Padova, Via Marzolo 1, Padova 35131, Italy; c Chair for Material and Surface Analysis, University of Wuppertal, Wuppertal 42119, Germany; d Department Chemistry, 14309 University of Cologne, Cologne 50939, Germany

**Keywords:** 2D perovskite, odd−even effect, photoluminescence, anisotropy, transport, photon reabsorption

## Abstract

Two-dimensional perovskites have emerged as promising materials for optoelectronic applications owing to their excellent environmental stability and tunable quantum confinement. Such 2D perovskites can incorporate a particularly versatile range of organic cations of different size, chemical nature, and optoelectronic character. However, understanding and controlling thin-film transport for this vast family of materials remains a key challenge to their successful application in devices. Here, we systematically investigate odd–even effects in thin films of Ruddlesden–Popper-type (RP) lead-iodide 2D perovskites based on nonconjugated alkylammonium spacer cations with chain lengths ranging from three to eight carbon atoms. A pronounced odd–even dependence on the carbon number is observed in both optical and transport properties, including absorption coefficients, photoluminescence energies and lifetimes, and excitation diffusion dynamics. Notably, the coefficients for charge-carrier diffusion out of the film plane—extracted via a dynamic photon reabsorption approach—display an opposite odd–even trend to the in-plane charge-carrier mobility obtained from optical pump–terahertz probe measurements, causing a pronounced odd–even modulation of the thin-film mobility anisotropy. Grazing-incidence wide-angle X-ray scattering measurements reveal that this behavior is related to cation-controlled nanostructural orientation: even-numbered alkyl spacer cations induce lead-iodide planes lying highly oriented within the film plane, while odd-numbered ones cause more disordered stacking. Furthermore, the observed 1/d^2^-dependence on interplane distance *d* in ordered films demonstrates that Förster resonance energy transfer underpins diffusion of excitations between lead-iodide layers. Our findings establish a direct structure–transport correlation in 2D perovskite films and provide valuable guidelines for the design of optoelectronic devices.

## Graphical abstract

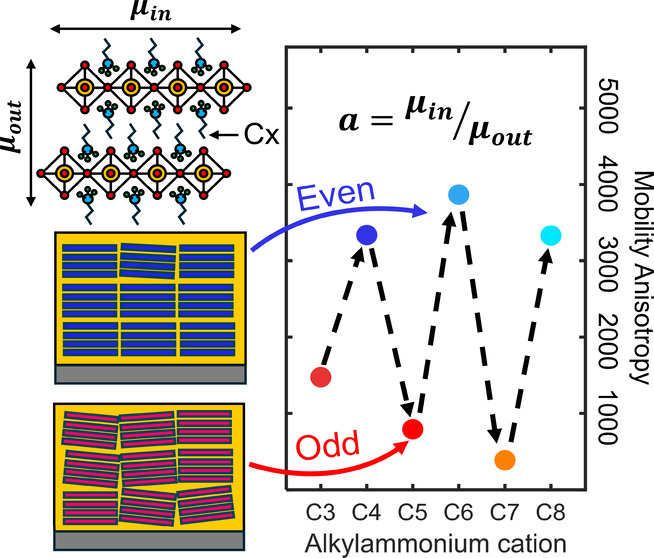

## Introduction

In recent years, metal halide perovskites (MHPs) have attracted tremendous attention in photovoltaic,^1,2^ light-emitting,^3,4^ and laser applications.^5,6^ Their remarkable optoelectronic properties include high charge-carrier mobility,^7^ low exciton binding energy at room temperature,^8^ and long photoluminescence (PL) lifetimes,^9^ rendering them excellent candidates for light-absorbing layers in solar cells. However, while power conversion efficiencies of MHP-based solar cells have reached 27.2% for single-junction devices,^10^ the reduced environmental and operational stability of some members of this semiconductor group remain a critical challenge, limiting their practical deployment.^11^

To address this issue, two-dimensional perovskites (2DPs) have been explored, either as part of 2D:3D perovskite heterostructures,^12–14^ or as the sole absorber layer^15^ in photovoltaic devices. 2DPs consist of inorganic metal-halide perovskite octahedral layers separated by large organic cation layers.^16^ Such layering facilitates considerable chemical tunability that opens a vast compositional space, where, in particular, hydrophobic large cations are used to enhance moisture resistance.^17^ Owing to the electronically insulating nature of these cations, strong quantum and dielectric confinement effects are imposed onto the perovskite layers, which redefines the photophysics of these 2DP materials compared to their 3D counterparts^18,19^ and opens further applications in light-emitting^3,4,20^ and quantum applications.^21^ Charge-carrier transport, in particular, is impacted by the increased excitonic nature and by the strongly anisotropic character of 2DPs.^22,23^ Numerous strategies have been developed to engineer charge-carrier transport in 2DPs, including the application of high external pressure,^24^ the incorporation of large electro-active cations,^25–30^ and the use of surface passivation layers.^31^ Despite the reported advances, several challenges related to the anisotropic nature of charge-carrier transport in these materials remain. One particularly acute challenge is the accurate determination of out-of-plane mobilities of charge carriers, which have been reported to be several orders of magnitude lower than those for in-plane transport in 2DPs.^22,32,33^ A variety of experimental techniques—such as optical pump–terahertz probe (OPTP) spectroscopy,^23,34,35^ PL-based mean square displacement (MSD) analysis,^36,37^ space-charge-limited current (SCLC) measurements,^32^ and microwave conductivity^33,38^ —have been employed to investigate transport processes in 2DPs. However, the aligned nature of 2DPs in thin films, as well as the strong transport anisotropies, mean that such techniques are often sensitive to only the dominant in-plane charge-carrier transport. While in thin films targeted for device fabrication, 2DP often form with layers strongly oriented parallel to the film plane,^39,40^ structural disorder may lead to partial loss of such alignment that can affect recorded mobilities normal to the film plane.^22^

To enable the rational development of large cation engineering in 2DPs and pursue the enhancement of charge-carrier transport in these materials, an accurate quantification of anisotropic transport is urgently required. Tuning such anisotropies is a major goal for exploitation of these materials, as desired magnitudes may vary strongly, depending on the intended charge-extraction or injection configuration and type of application. However, the clean separation of in-plane and out-of-plane diffusion of charge carriers remains a challenge, in particular in thin films of 2DPs that are relevant to device applications.^41^ Recently, we reported a PL-based approach that exploits a dynamic photon reabsorption process to track out-of-plane excitation diffusion in polycrystalline films of the 2DP PEA_2_PbI_4_.^22^ This approach paves the way for investigating how out-of-plane diffusion coefficients and the resulting transport anisotropy can be engineered through the choice of the organic spacers. Recent studies have shown that varying the alkyl spacer length in layered perovskites can give rise to a pronounced odd–even effect in their structural, optical, and electrical properties, as reported both by other groups and in our previous work.^42–44^ These earlier findings have identified spacer parity as an important design parameter in 2DP thin films, but its influence on out-of-plane diffusion and transport anisotropy has not yet been directly quantified.

In this work, we systematically investigate both in-plane and out-of-plane diffusion of excitations in thin films of lead-iodide 2DPs with organic alkyl spacer cations containing three to eight carbon atoms. For the Ruddlesden–Popper (RP) type 2DPs employed, highly oriented films are formed with octahedral monolayers (*n* = 1) parallel to the film plane. We observe a pronounced odd–even trend in the optical properties of these films, including the absorption coefficient, PL energy and lifetime. Importantly, diffusion coefficients associated with charge-carrier motion out of the film plane, obtained through our previously reported photon reabsorption method,^22^ are found to be lower for even-numbered alkyl cations than for odd-numbered ones. Intriguingly, this odd–even trend is opposite to that observed for the in-plane charge-carrier mobility measured by OPTP.^43^ As a result, the charge-carrier anisotropy ratio in the films follows a particularly pronounced odd–even trend, revealing a strong tunability with alternating carbon chain length of the organic spacer. Through grazing incidence wide-angle X-ray scattering (GIWAXS) analysis, we reveal that the strong odd–even variation of diffusion out of the film plane originates from changes in the nanostructural layer orientation with carbon chain length. While 2DP films with even-numbered carbon chains form with lead-iodide layers highly oriented within the film plane, resulting in strong transport anisotropy, those with odd-numbered spacers show higher local orientational disorder leading to faster charge motion out of the film plane. In addition, we reveal that interplane diffusion of excitations in these materials predominantly proceeds via the Förster resonance energy transfer (FRET) mechanism, based on the observation of a 1/d^2^-dependence of diffusion coefficients on the interplanar distance. Overall, our findings uncover the peculiar odd–even character of charge-carrier transport and anisotropy in 2DP films, thus providing valuable insights for the rational design and optimization of 2DP-based optoelectronic devices.

## Results and Discussion

We studied a series of RP type lead-iodide 2DP films incorporating linear alkylammonium cations of increasing length, ranging from *n*-propylammonium (C3) to *n*-octylammonium (C8) ((Cx)_2_PbI_4_, see Figure 1a, and Table S1 for full chemical formulas). In the n = 1 Ruddlesden–Popper structures, the inorganic [PbI_6_]^4–^ corner-sharing octahedral layers are separated by double layers of large alkyl ammonium cations. The spacing between the inorganic perovskite layers (henceforth referred to as interlayer distance, *d*) increases linearly with the carbon-chain length of the large cation. The variation in interlayer distance and film quality was verified by X-ray diffraction (XRD) measurements (Figure S1) and GIWAXS analysis, which indicated highly preferential orientation of layers parallel to the film and substrate plane (Figures S30 - 35). The absorption spectra (Figure 1b) reveal a distinct odd–even trend in excitonic peak positions: the exciton peaks of odd-numbered cations (red-colored series) exhibit a continuous redshift from 492 to 508 nm as the carbon-chain length increases, whereas those of even-numbered cations (blue-colored series) remain nearly unchanged. As reported previously, this observation indicates that the bandgap of the odd-numbered 2DP films decreases with increasing spacer length, while that of even-numbered counterparts remains nearly constant.^43^ The PL peak positions exhibit the same behavior as the absorption spectra – C3, C5, and C7 films show emission peaks at 501, 511, and 518 nm, respectively, whereas C4, C6, and C8 films all emit around 522 nm – confirming the presence of a pronounced odd–even modulation in bandgap energy. This phenomenon has been attributed to variations in the Pb–I–Pb bond angle within the inorganic layers, induced by the distinct packing configurations of the large organic cations.^43^ It is worth noting that the films investigated here do not show evidence of significant phase impurity within the resolution of our measurements. Based on the PL spectra (Figure 1c), absorption (Figure 1b), and XRD results presented (Figure S1), we only observe signatures consistent with the expected RP phase in these lead-iodide 2D perovskite films. In particular, the XRD patterns are consistent with layered RP-type ordering, and the PL and absorption spectra agree well with the characteristic optical features of RP-phase 2D lead-iodide perovskites.

**Figure 1:**
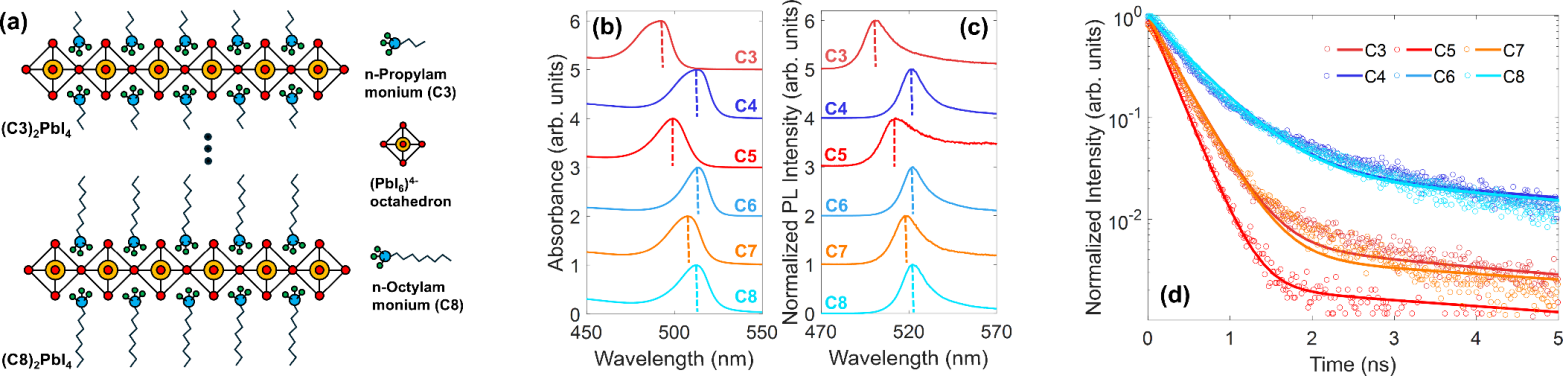
(a) Illustration of the crystal structure of *n* = 1 Ruddlesden–Popper (C*x*)_2_PbI_4_ 2DPs incorporating a series of nonconjugated alkylammonium spacer cations, ranging from *n*-propylammonium (C3) to *n*-octylammonium (C8). (b) absorption spectra, (c) steady-state PL spectra, and (d) TRPL transients of 2DP thin films with various alkylammonium spacer cations collected by TCSPC. PL was recorded following excitation with a laser of 398 nm wavelength. Solid lines in (d) are fits based on biexponential fitting (see section 5.3 in SI).

Interestingly, we here find that a distinct odd–even dependence is also observed in the lifetimes of photoluminescence, as revealed by time-resolved PL (TRPL) measurements (Figure 1d). PL transients are dispersive, showing an initial fast component, followed by a slower decay. We extract an average PL lifetime value (see Section 5.3 and Table S2 in Supporting Information for analysis) for all 2DP films and find that while for odd-numbered alkyl chains this is consistently around 0.32 ns, for even-numbered chains a significantly longer lifetime of 0.62 ns is observed. We ascribe the extended PL lifetimes for 2DP films with even-numbered spacers to improved material crystallinity, given that in metal halide semiconductors, nonradiative recombination centers and defects have been shown to accumulate preferentially near grain boundaries.^45,46^ Our hypothesis is supported by the OPTP and GIWAXS analyses discussed in detail further below, which reveal enhanced structural crystallinity in films containing even-numbered spacers. As recently demonstrated, such dependence of film morphology on carbon number is closely linked to the distinct molecular packing modes of the organic spacer cations.^42,43^ We note that the possible influence of surface morphology has also been considered. Top-view SEM images (Figure S9) indicate that the film surface morphology is broadly similar across the series and does not show a systematic odd–even variation. Therefore, the observed differences in PL lifetimes are unlikely to originate from morphological variations alone.

To reveal the effect of these photophysical changes within the 2DP series on charge-carrier transport, we employed two different measurement probes that determine charge diffusion either in the direction normal to the thin film plane, or within the plane. In this work, we use the term excitations as a general description of the photoexcited population in these 2D perovskite films. This choice is motivated by previous OPTP measurements, which showed that even in 2D perovskites with relatively high exciton binding energies, a finite charge-carrier population can coexist with excitons at room temperature, with a charge-carrier-to-exciton ratio of close to 0.3 under relevant conditions.^23^ Similar conclusions have also been noted by other studies.^47,48^ Therefore, we use excitations here as a more general term that does not imply the exclusive presence of either free carriers or excitons alone. We first examined diffusion of excitations out of the film plane, based on the previously reported photon reabsorption method.^22,41^ In this method, films are excited with laser pulses from one side (here the side of the film facing air), and PL spectra are recorded over time both for emission toward the same side and for emission toward the opposite side with respect to where excitation had occurred. Because charge carriers are initially generated close to the excitation surface, for the former geometry (same-side collection, air-side excitation, SA) fewer emitted photons are reabsorbed by the material than for the latter geometry (opposite-side collection, air-side excitation, OA). However, as charge carriers diffuse through the film depth profile and their density becomes evenly distributed, both geometries approach similar photon reabsorption effects. Since photon reabsorption affects mostly the high-energy end of the spectrum which overlaps prominently with the absorption spectrum, for the SA geometry, increasing self-absorption effects lead to a red shift over time, while for the OA geometry, weakening self-absorption causes a mirroring blue shift. We have shown that by modeling such dynamic effects, out-of-plane diffusion in 2DP films can be accurately quantified.^22^ Figure 2 presents examples of such transient PL spectra for films of lead-iodide 2DPs based on either the *n*-Butylammonium (C4) or the *n*-Pentylammonium (C5) cation, for both the SA and OA configurations, recorded using an intensified CCD (iCCD) at times between 1 and 60 ns after excitation (see Figures S10–13 for other 2DPs). Because the PL lifetimes of these 2DP films are relatively short—as shown in Figure 1d—we focus our analysis on the spectral evolution within 60 ns. We find that indeed the PL spectra exhibit a progressive redshift with increasing delay time for the SA geometry (Figures 2a, d) and a matching blueshift in the OA geometry (Figures 2b, e), consistent with changes in photon reabsorption as the initially generated excitations propagate away from the front (air) surface and deeper into the film.

**Figure 2:**
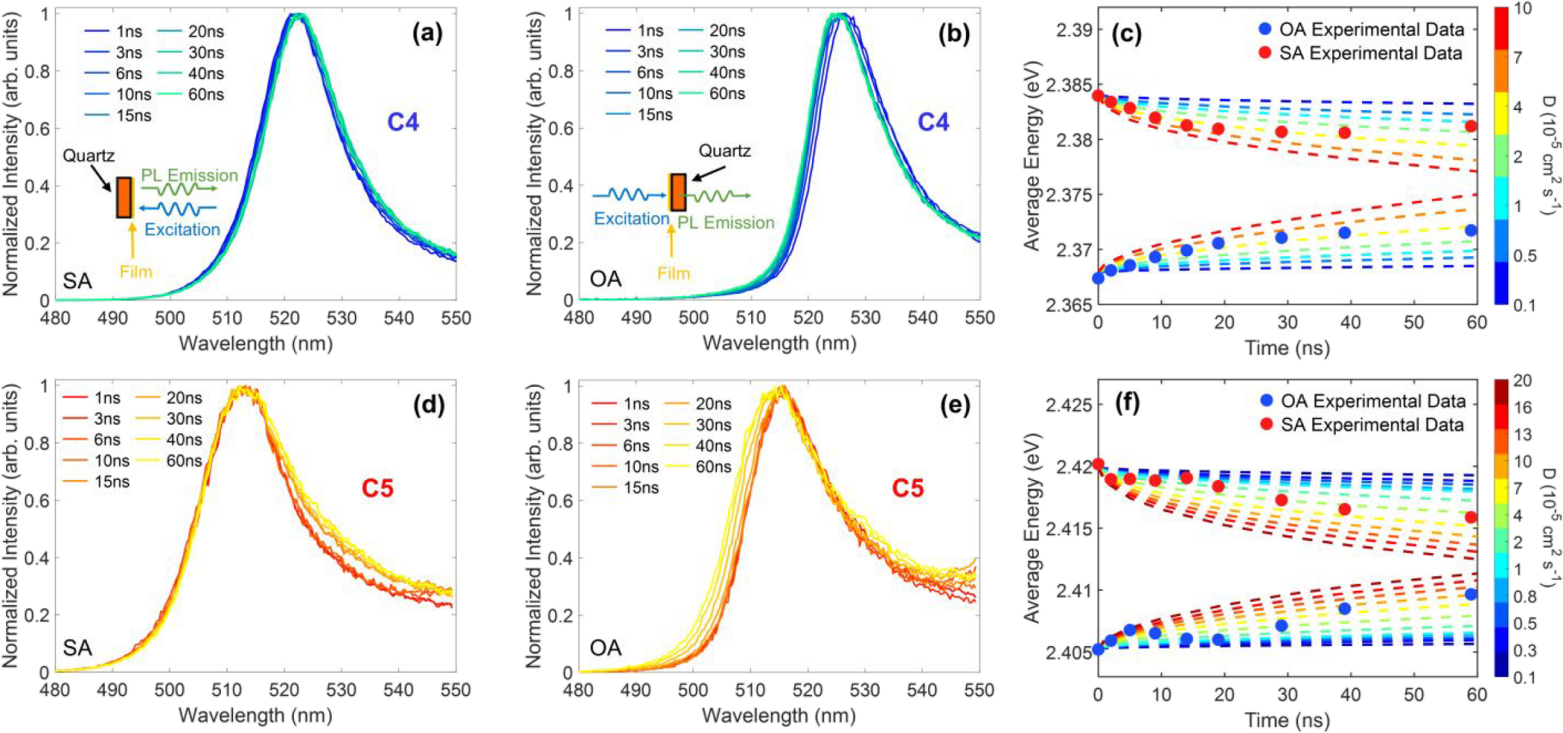
Photon reabsorption-based measurements of out-of-plane diffusion of excitation along the depth profile of 2DP thin films. (a) and (b) show transient PL spectra for a thin film of the C4-based 2DP (C_4_H_9_NH_3_)_2_PbI_4_ collected at times ranging between 1 and 60 ns after excitation with 398 nm light pulses, for SA and OA configurations, respectively. Insets: schematics of the SA and OA measurement configurations. (c) Average photon energy (dots) and simulated results (dashed lines) for C4-based films for SA and OA configurations (average energy calculation window: 460 nm – 530 nm). (d) and (e) show transient PL spectra for a thin film of the C5-based 2DP (C_5_H_11_NH_3_)_2_PbI_4_ collected at times ranging between 1 and 60 ns after excitation with 398 nm light pulses, for SA and OA configurations. (f) Average photon energy (dots) and corresponding simulated results (dashed lines) for C5-based films for SA and OA configurations (average energy calculation window: 460 nm – 520 nm).

In order to extract coefficients for diffusion out of the film plane, we first quantify these spectral shifts, by defining the average energy as

$$\left\langle E\right\rangle =\frac{\int f_{\left(E\right)}\cdot EdE}{\int f_{\left(E\right)}dE}$$ 1

where *f*_(*E*)_ represents the PL line shape as a function of energy *E*. By definition, a decrease or increase in ⟨E⟩ correspond to a red or blue shift, respectively. The temporal evolution of such extracted average energy values is shown in Figure 2c,f, for SA and OA configurations (red and blue dot symbols), recorded for 2DP films incorporating either C4 or C5 spacers (see SI Figures S10–S13 for other cations). For both 2DPs, the two curves gradually approach each other with increasing delay time, indicating that the gradual leveling of the charge density across the complete film profile leads to an equilibration of self-absorption effects^22^ which evolves over a time scale of ∼100 ns.

To extract diffusion coefficients for charge motion along the direction of the film depth profile from the dynamic evolution of ⟨E⟩, we simulated the expected changes in PL spectra using a one-dimensional diffusion model (for a detailed description of the procedure, see Section 6 of the SI). We note that these 2DPs show a low-energy emission peak typical of such materials, which has been observed previously and attributed to a trap state caused by local compositional variations or precursor depletion during film growth.^49^ However, we ensure that such emission does not influence the extraction of diffusion coefficients from the photon reabsorption method by (i) selecting the spectral emission region dominated by the intrinsic free-charge and exciton emission (ii) analyzing spectra over the first 60 ns where low-energy emission peaks are not yet dominant, (iii) ensuring that average energy data exhibit symmetric red- and blue-shifts when the detection side is changed from the same (SA) to the opposite (OA) side with respect to the excitation, and (iv) observing that, in our lead-iodide 2D perovskite thin films incorporating alkyl organic spacer cations, the relatively small Stokes shift results in the low-energy emission peak being spectrally well separated from the corresponding absorption edge (see Section 6.5 of the SI for a full discussion). In our simulations, we use an extrapolated expression for the intrinsic emitted spectra, and assumed a surface recombination velocity of 1.18 × 10^4^ cm s^–1^ at the air side of the films (see Section 6.2 of the SI), which is comparable to previously reported values for 2DP films.^22^ As shown by the colored dashed lines in Figure 2c,f for the examples of (C4)_2_PbI_4_ and (C5)_2_PbI_4_, the experimentally measured ⟨E⟩ curves are in good agreement with specific simulated curves, thus allowing us to determine an out-of-plane diffusion coefficient (see Section 6.4 for details) of (0.39 ± 0.04) × 10^–4^ cm^2^ s^–1^ for the 2DP based on C4, and (0.6 ± 0.1)×10^–4^ cm^2^ s^–1^ for C5. Such low values are generally consistent with the strong preferential alignment of the lead-iodide octahedral layers within the film plane. While interlayer transfer of excitations is slow, in agreement with our previous report on phenylethylammonium (PEA)-based 2DPs,^22^ in-plane diffusion coefficients are typically on the order of 10^–1^ cm^2^ s^–1^,^36,37^ resulting in a strongly anisotropic character of charge-carrier transport in these materials. We further note that while for the C4-based 2DP film, experimentally determined ⟨e⟩ values for both SA and OA geometries fluctuate only slightly around a single simulated curve over time (Figure 2c), for C5 the data exhibit more pronounced fluctuations and nonmonotonous evolution (Figure 2f). As discussed in the mechanism section below (Figure 4) and our previous work, such nonmonotonous evolution can be indicative of variations in nanostructural orientation across the 2DP thin film depth profile.^22^ As such, we may therefore use the statistical variations between experimental and simulated ⟨E⟩ transients, reflected in the standard error of the diffusion coefficient (Figure S19), as a proxy for the extent of orientational disorder in the 2DP films.

To examine systematically how the length of the alkyl spacer chain affects diffusion of excitations in the direction normal to the film plane, we analyzed the dynamic photon reabsorption effect and extracted diffusion coefficients for the entire series of 2DP films by computing the mean of the time-dependent diffusion coefficient, following the procedure outlined above (see Section 5 and 6 of SI). For each delay time, the experimental average-energy shift was compared with the simulated result to determine the corresponding diffusion coefficient D. The diffusion coefficient reported for each film is obtained as the mean value of the time-dependent D values derived over the full measured temporal range (full details in Section 6.4 of SI). Figure 3a displays the mean out-of-plane diffusion coefficients extracted for 2DPs with alkylammonium chains ranging from C3 to C8. Intriguingly, these data reveal a distinct odd–even trend across the 2DP thin film series: 2DP films based on spacer cations with odd-numbered carbon chains (C3, C5, C7) show consistently higher coefficients for diffusion perpendicular to the film plane compared to those with even-numbered (C4, C6, C8) spacers. This odd–even effect seems counterintuitive at first, given that energy and charge transfer rates are expected to simply decrease monotonically as the interlayer distance increases with larger spacer cations.^50^ Typically, the lead-halide octahedral planes are strongly aligned within the film plane, therefore, the out-of-planes diffusion we record here might initially be assumed to mostly reflect such interplane transfer of excitations. However, as an important clue to the mechanism underpinning such odd–even effects, we note that the standard errors of the extracted diffusion coefficients also exhibit an odd–even dependence, with larger variations found for the odd-numbered spacer chains (Figure S19). As mentioned above and shown previously,^22^ such fluctuations suggest a stronger presence of local orientational disorder or structural inhomogeneity associated with the packing of odd-numbered organic spacers, which may in turn affect the diffusion normal to the film plane in these 2DP films. The same structural disorder may also introduce an additional in-plane contribution to the experimentally extracted out-of-plane diffusivity, which can make the values for the odd-numbered spacer films appear systematically larger than their intrinsic out-of-plane transport would suggest, as discussed in detail further below. We note that sub diffusion has previously been reported in layered perovskites, particularly in measurements where trap states lead to a time-dependent slowing of excitation diffusion on the nanosecond time scale.^36^ In the present work, however, we do not observe evidence of such behavior within the experimental time window studied here. Instead, the diffusion coefficients extracted at different delay times remain close to a single value within experimental uncertainty, in particular for even-numbered carbon chains, indicating that the measured average-energy dynamics can be adequately described by the one-dimensional normal-diffusion model used in our analysis (see detailed discussion in Section 6.4 of SI).

**Figure 3:**
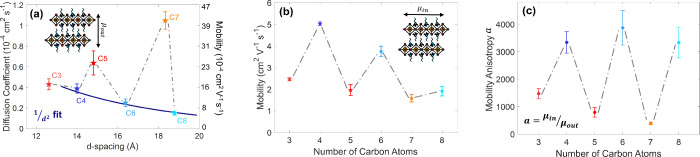
Odd–even trends in out-of-plane and in-plane excitation diffusion for the C3–C8 series of (C*x*)_2_PbI_4_ 2DP films. (a) Out-of-plane diffusion coefficients of excitations (diffusion coefficients are reported as the mean of the time-dependent D values extracted over the measured temporal range), extracted using the photon reabsorption method, along with a fit based on a 1/*d* ^2^ dependence (blue line) applied to films with even-numbered spacer cations (C4, C6, and C8). (b) In-plane effective electron–hole sum mobility obtained from OPTP measurements. (c) Mobility anisotropy ratio *a* between in-plane and out-of-plane diffusion.

We further investigated the in-plane transport properties of these films by using THz conductivity spectroscopy, in which the electric field polarization of the incident THz wave is polarized in the direction parallel to the film and substrate. Because the 2DP layers are also highly oriented within the film plane, such measurements predominantly probe the mobility of charge carriers within the lead-iodide octahedral planes.^23,43^ Figure 3b presents in-plane charge-carrier mobilities extracted from OPTP measurements through linear fits to the excitation-fluence–dependent terahertz transmission (full details provided in Section 7 of SI). Importantly, while a clear odd–even trend with alkyl chain length of the cations is again observed for in-plane mobilities (Figure 3b), the dependence is inverse to that found for out-of-plane mobilities (Figure 3a). As discussed in our previous work,^43^ such higher in-plane mobility for 2DPs with even-numbered spacer cations compared to those with odd-numbered spacers, is directly linked to differences in molecular packing efficiency between odd and even large spacer cations, which in turn lead to changes in Pb–I–Pb bond angles. This templating effect on the lead-iodide lattice thus causes odd–even modulations in octahedral distortions that will affect band-edge dispersions, electron–phonon coupling and therefore charge transport within these inorganic layers.^43^

From the combination of in-plane and out-of-plane transport measurements we are further able to assess the charge-transport anisotropy of 2DP films as a function of alkylammonium spacer length. To enable a direct comparison between the two types of measurements, we converted the derived out-of-plane diffusion coefficients to effective mobilities using the Einstein relation, as indicated on the secondary (right) *y*-axis of Figure 3a. We note that the two experimental methods are not sensitive to the same excited-state species to the same extent: OPTP measurements predominantly probe mobile charge carriers, whereas the reabsorption-based PL method probes PL-active excitations responsible for the time-dependent spectral shift, which may include both radiatively decaying excitons and free electron–hole pairs. However, these excited-state populations are likely to remain dynamically coupled through mutual interconversion,^23^ so that their qualitative trends can still be influenced by the same underlying structural factors, such as film orientation and nanostructural order.

The derived in-plane to out-of-plane mobility anisotropy factor—defined here as *a* = μ_in_/μ_out_—follows a particularly pronounced odd–even trend along the C3–C8 series (see Figure 3c), as a result of the counteracting trends of the two components. The overall magnitude of the transport anisotropy is on the order of ∼ 10^3^, with odd-numbered spacer chains reaching anisotropies near 1000, while values above 3000 are found for even-numbered chains. These results collectively reveal that the parity of the organic spacer has a remarkably strong effect on the anisotropy of excitation transport in 2DP films. These anisotropy values are consistent with the strongly layered nature of 2D perovskites, in which transport is expected to proceed much more efficiently within the inorganic octahedral slabs than across the organic spacer layers.^41,51^ Importantly, similarly large anisotropies have also been reported previously using other experimental methods. For example, Yun et al. reported in-plane and out-of-plane mobilities of 1.2 cm^2^ V^–1^ s^–1^ and 1.5 × 10^–4^ cm^2^ V^–1^ s^–1^, respectively, for layered metal halide perovskites measured by the SCLC method, corresponding to an anisotropy of approximately 8000.^32^ Fei et al. likewise observed strongly suppressed out-of-plane transport in 2D perovskite films using TRMC measurements.^33^ Therefore, our results fall well within the range expected for strongly anisotropic layered perovskites.

We further show that the inverse odd–even effect observed for out-of-plane diffusion is directly linked with increased orientational disorder in films of 2DPs with odd-numbered carbon chains. We performed GIWAXS measurements with incident angles ranging from 0.05° (probing the air-perovskite interface) to 0.20° (penetrating through the entire film) (details in Section 8 of the SI). Figure 4a,b shows the (002) diffraction patterns obtained for (C4)_2_PbI_4_ and (C5)_2_PbI_4_ films, respectively. For the even-numbered spacer (C4), the narrow reflections observed in the diffraction pattern reveal a highly oriented nanostructure with minimal contribution from large azimuthal angles, thereby indicating well-aligned crystalline domains with octahedral layers lying parallel to the substrate and film plane. In contrast, for C5 (odd-numbered spacer) the GIWAXS data Exhibit significantly broader features, extending along semicircular arcs, thus implying noticeable nanostructural and orientational disorder. Crucially, such disorder in the alignment of the lead-halide octahedral planes with respect to the substrate means that charge-carrier motion in the direction normal to the film (out-of-plane) may now have significant contributions from motion occurring along the lead-iodide octahedral planes.^22^ Owing to the significant intrinsic transport anisotropy in 2DPs—with mobility within octahedral planes being ∼3 orders of magnitude higher than mobility normal to the planes—even such slight misalignment of these layers out of the film plane can enhance the transport in the direction normal to the film plane substantially. As a result, the diffusion coefficients recorded for motion normal to the film plane (Figure 3a) reveal much higher values for 2DPs with odd-numbered carbon chains for which such orientational disorder is significant.

**Figure 4:**
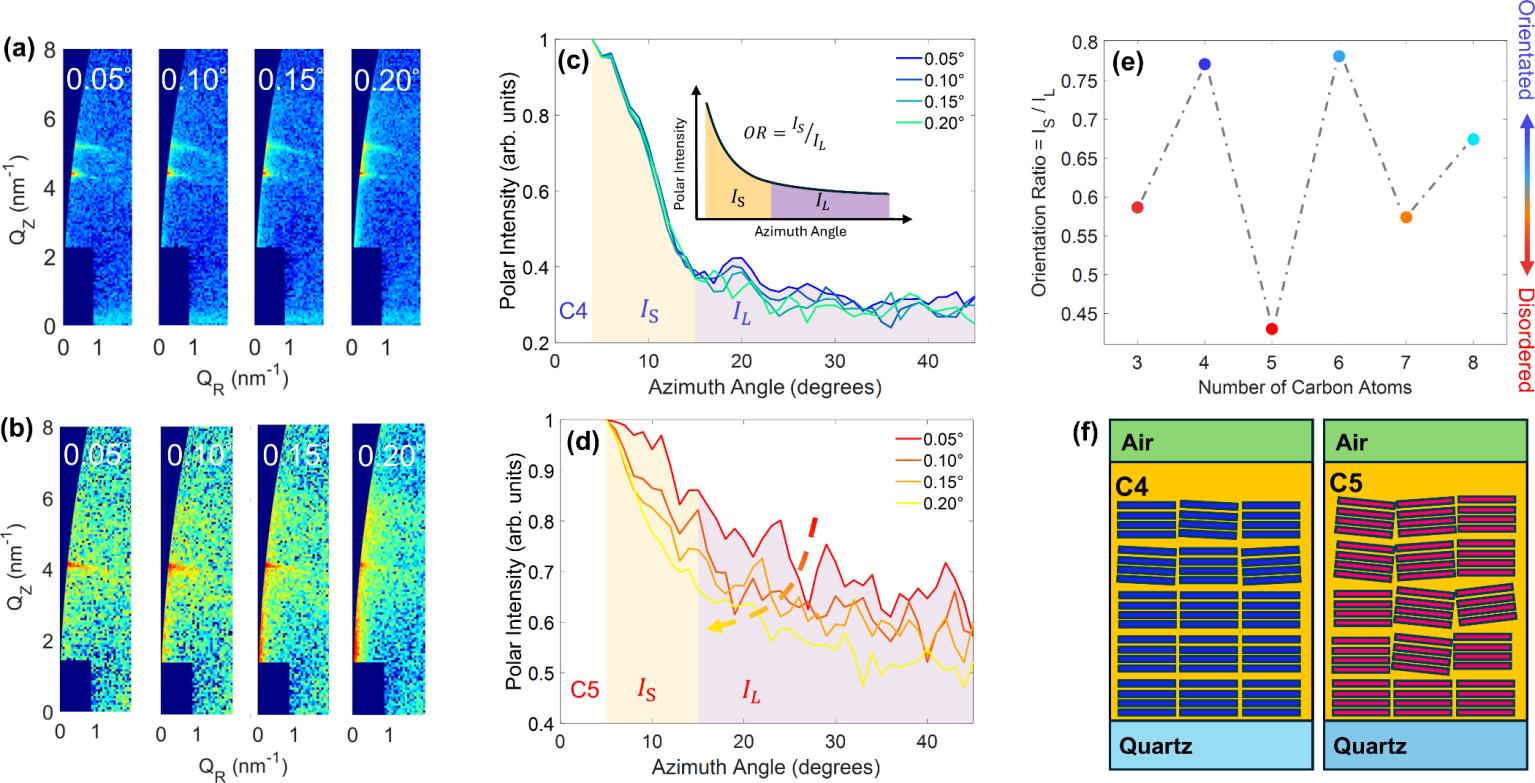
GIWAXS characterization of nanostructural orientation in (C*x*)_2_PbI_4_ 2DP films. (a) and (b) Depth-dependent GIWAXS patterns recorded for C4- and C5-based 2DP films. The intensity of each figure has been normalized separately. (c) and (d) Polar intensity profiles of the (002) diffraction peak collected at incident angles ranging from 0.05° (probing the film surface) to 0.20° (penetrating through the entire film). Inset in (c): Schematic illustration of definition for the orientation ratio (OR). (e) OR values for the C3–C8 series of (C*x*)_2_PbI_4_ 2DP films at an incident angle of 0.05°. (f) Schematic illustration showing the difference in crystalline layer orientation with respect to the substrate plane and nanostructural ordering between films of (Cx)_2_PbI_4_ with C4 (even-number) and C5 (odd-number) spacer groups.

To compare quantitatively the nanostructural layer orientation in films of (C*x*)_2_PbI_4_ with even- and odd-numbered carbon chains, we analyzed the azimuthal polar intensity distributions (0–45°) in GIWAXS data recorded at varying incident angles (see Figure 4c,d). For (C4)_2_PbI_4_ films, the intensity profiles remain nearly constant across different incident angles, with the normalized intensity between 15° and 45° remaining around 0.3 (Figure 4c), confirming uniform crystalline orientation throughout the film. Conversely, (C5)_2_PbI_4_ films exhibit a pronounced decrease in the normalized polar intensity at larger azimuthal angles (15° to 45°) with increasing incident angle, indicating progressive loss of orientation in the film from the substrate toward the air interface (Figure 4d). We further quantified such effects by determining an orientation ratio (OR), which describes the degree of nanostructural layer alignment (inset of Figure 4c) as follows:

$$OR=\frac{I_{S}}{I_{L}}$$ 2

where *I*_*S*_ and *I*_*L*_ are the integrated intensities over azimuthal angles of 0–15° (indicated by yellow background) and 15–45° (indicated by purple background), respectively. A higher value of OR thus indicates a greater degree of layer orientation parallel to the substrate. Figure 4e summarizes the OR values of the (002) diffraction peaks for all (C*x*)_2_PbI_4_ films (C3–C8) measured at an incident angle of 0.05°. Importantly, a clear odd–even dependence on the large cation chain length is revealed. Here, films with even-numbered spacers exhibit significantly larger OR values than their odd-numbered counterparts, thus confirming that even-numbered large cations yield more ordered layer orientation (as illustrated by the double arrow in Figure 4e) than those with odd-numbered cations.

Based on these results, we propose the structural model illustrated in Figure 4f. (C*x*)_2_PbI_4_ 2DP films based on odd-numbered cations (C3, C5, C7) possess more disordered layering, thereby allowing charge-carrier motion in the direction normal to the film plane to be assisted by motion along misoriented lead-iodide octahedral planes. Since transport along lead-iodide octahedral planes is around 3 orders of magnitude faster than between lead-iodide planes, even such slight misalignment considerably increases charge diffusion along the film depth profile, and the transport anisotropy for thin films with odd-numbered carbon chains is thus substantially lowered. In contrast, (C*x*)_2_PbI_4_ with the even-numbered carbon chains (C4, C6, C8) exhibit highly oriented nanostructures, for which the measured diffusion out of the film plane accurately reflects the intrinsic transport normal to the lead-iodide octahedral layers. This structural origin explains the odd–even effect observed for the out-of-plane diffusion coefficients shown in Figure 3a. Our structural characterization also explains the observed odd–even trends in the statistical fluctuation associated with the extracted values for out-of-plane diffusion coefficients (Figure S19 and error bars in Figure 3a). The larger uncertainties for the films with odd-numbered carbon chains indicate stronger temporal fluctuations in the extracted diffusion coefficients caused by such structural and orientational disorder, whereas the smaller uncertainties for the even-numbered films reflect uniform orientation and reduced temporal variability in the diffusion process. Therefore, the experimentally extracted out-of-plane diffusivity is not governed by spacer length alone, but instead reflects the interplay of two contributing factors. The first is the intrinsic spacer-length effect governed by the Förster resonance energy transfer (FRET) mechanism: as the organic spacer becomes longer, the interlayer distance increases and the intrinsic out-of-plane transport is therefore expected to decrease.^50^ The second factor is the effect of nanostructural orientation. In films with structural misorientation, a partial in-plane contribution can add to the experimentally extracted out-of-plane diffusivity, leading to values that are larger than the intrinsic out-of-plane diffusivity of the material.^22^

These trends are indeed observed in the (C*x*)_2_PbI_4_ film series. The highly ordered films formed with even-numbered carbon chain spacers follow the FRET mechanism expected for interlayer transfer of excitations, with diffusion coefficients inversely proportional to the square of the interlayer spacing (1/d^2^). As the dark blue line in Figure 3a shows, for (C*x*)_2_PbI_4_ films with C4, C6, and C8 spacers, we find a satisfactory fit of a 1/d^2^ expression to the out-of-plane diffusion coefficients, further validating the proposed FRET transfer mechanism. In contrast, for the odd-numbered spacer films, the larger degree of nanostructural disorder introduces additional in-plane conductivity contributing to the experimentally extracted out-of-plane diffusivity. This additional contribution can obscure the intrinsic spacer-length dependence, explaining why the films comprising 2DPs with odd-numbered carbon chains do not follow the same trend as the those with even-numbered chains.

## Conclusions

In conclusion, we have systematically investigated both in-plane and out-of-plane excitations transport in thin films of Ruddlesden–Popper 2D lead-iodide perovskites by tuning the length of the alkyl chain in the spacer cations, from three to eight carbon atoms. A pronounced odd–even trend with the number of carbon atoms was observed in several optical and transport properties, including steady-state photoluminescence, absorption, PL lifetime, and excitation transport both in and out of the thin-film plane. Coefficients for diffusion of charge carriers out of the film plane, extracted using a dynamic photon reabsorption-based method, exhibit an opposite odd–even trend to the in-plane charge-carrier mobility obtained from OPTP measurements. GIWAXS analysis revealed that for out-of-plane diffusion, such odd–even effects originate from parity-dependent changes in nanostructural layer orientation: even-numbered spacer cations promote highly oriented domains with enhanced anisotropy and reduced diffusivity of charges along the film depth profile, whereas odd-numbered cations induce more disordered stacking and faster diffusion out of the film plane. Such slight misalignment of layers with respect to the substrate plane substantially accelerates diffusivity out of the film plane because of the high intrinsic anisotropy of in-layer to interlayer transport in these 2DPs (around 3 orders of magnitude). For charge-carrier mobility within the film plane, on the other hand, odd–even effects are related to changes in molecular packing efficiency that template the lead-halide octahedra, in turn affecting intralayer mobilities.^43^ Taken together, the two opposing trends lead to a particularly pronounced odd–even modulation in the thin-film charge transport anisotropy of the (C*x*)_2_PbI_4_ films. Finally, it is shown that for the highly oriented series of (C*x*)_2_PbI_4_ films with spacers containing even-numbered carbon chains, the excitation transport between lead-iodide layers follows a 1/d^2^ dependence on layer spacing, confirming that this proceeds predominantly via the Förster resonance energy transfer mechanism. Overall, these findings highlight the fundamental influence of organic spacer parity on thin-film transport and its anisotropy in 2D perovskites, providing valuable design principles for optimizing their optoelectronic performance in next-generation devices.

## Methods

### Sample Fabrication

For this study of two-dimensional (C*x*)_2_PbI_4_ perovskites, a series of alkylammonium iodide salts (C_*x*_H_2*x*+1_NH_3_I), as listed in Table S1 in the SI, were employed. The carbon chain length of the spacer cations ranged from *x* = 3 (*n*-propylammonium iodide, labeled as C3) to *x* = 8 (*n*-octylammonium iodide, labeled as C8). The alkylammonium iodide salts corresponding to C3–C6 and C8 were purchased from TCI, while *n*-heptylammonium iodide (C7) was synthesized as described below. The precursor solutions for the 2D perovskite films were prepared by dissolving lead iodide (PbI_2_, TCI) and the organic cation salts in dimethylformamide (DMF, Sigma-Aldrich) with a molar ratio of PbI_2_:(C*x*)I = 1:2. The precursor solutions (0.3 M) were heated and stirred at 50 °C for 3 h prior to film deposition. Thin films were then fabricated by spin-coating the precursor solutions for 40 s (with a 3 s ramp). To achieve a consistent film thickness among the samples, the spin-coating speeds were optimized for each film, ranging between 3000 and 5000 rpm. The films were subsequently annealed at 100 °C for 10 min. All solution preparation and film fabrication processes were conducted under a nitrogen atmosphere.

### Synthesis of *n*-Heptylammonium Iodide (C7)

The *n*-heptylammonium iodide was synthesized by protonation of the corresponding primary amine with aqueous hydriodic acid. An optimized low-temperature synthesis strategy was employed to enable controlled protonation and to improve the purity and reproducibility of the resulting ammonium iodide.^52^ Heptylamine (0.085 mol) was weighed into a flame-dried Schlenk-flask under an argon atmosphere. The solution was cooled to −78 °C while being magnetically stirred. Aqueous hydriodic acid (57 wt % in H_2_O, ρ ≈ 1.70 g mL^–1^, Sigma-Aldrich) was added dropwise over approximately 10 min (19.09 g, 11.2 mL, 0.085 mol, 1.0 equiv), while maintaining the temperature at −78 °C. After complete addition, the reaction mixture was allowed to warm slowly to room temperature and stirred for an additional 3 h. Solvent, water and excess HI were removed under reduced pressure on a rotary evaporator at 50 °C, affording a viscous residue. The crude product was triturated with ice cold diethyl ether (3 × 30 mL), and the supernatant was decanted after each washing step. The remaining solid was dried at 50 °C on a rotary evaporator and subsequently under high vacuum (∼24 h), yielding the corresponding heptylammonium iodide (C7) as a white hygroscopic solid. The salt was stored under dark and inert atmosphere and used without further purification.

### XRD Measurements

X-ray diffraction measurements (XRD) were measured using a Panalytical X-pert powder diffractometer. The Cu–Kα X-ray source (λ=1.54 Å) was set to 40 kV voltage and 40 mA current.

### Absorption Measurements

Absorption spectra were collected using a Fourier-transform infrared (FTIR) spectrometer (Bruker Vertex 80v) equipped with a xenon lamp source, a calcium fluoride beam splitter, and a silicon diode detector.

### Photoluminescence

Time-resolved PL measurements were performed using a gated intensified charge-coupled device (iCCD) to record PL spectra at defined time delays following photoexcitation. A 398 nm diode laser (PicoQuant LDH-D-C-398M) with a repetition rate of 1 MHz and a fluence of 115 nJ cm^–2^ was used to excite the perovskite thin films. The emitted PL was dispersed by a grating spectrometer (Princeton Instruments SP-2558) and subsequently detected by a silicon iCCD detector (PI-MAX4, Princeton Instruments). The excitation source and detection system were synchronized using a Keysight Technologies 33600A Trueform waveform generator. All PL measurements were conducted under vacuum conditions (∼3 × 10^–2^ mbar) by placing the samples inside a sealed vacuum chamber. For the measurements shown in Figure 1d, time-correlated single-photon counting (TCSPC) was used to record PL transients as a function of time after excitation, employing a 398 nm picosecond pulsed diode laser with a 1 MHz repetition rate. A PicoHarp 300 TCSPC event timer was used to synchronize and control the timing of photon detection.

### OPTP Measurements

OPTP measurements were performed using a Spectra Physics Mai Tai–Ascend–Spitfire Pro Ti:sapphire regenerative amplifier, producing 35 fs pulses at an 800 nm center wavelength and a 5-kHz repetition rate. THz probe pulses were generated using a spintronic emitter coated with antireflection and high-reflectivity layers.^53^ Samples were set in an evacuated chamber (pressure <10^–1^ mbar) and excited by the 400 nm optical pump, followed by probing with the THz pulse after a controlled delay. Pump and THz beams were modulated at 1.25 kHz and 2.5 kHz, respectively, using optical choppers to extract the pump-induced change in THz transmission (Δ*T*). Pump power was adjusted using a neutral-density filter wheel. THz transmission through the thin films was detected via electro-optic sampling in a 1 mm-thick (110)-oriented ZnTe crystal using a spatially and temporally overlapped 800 nm gate pulse. The THz signal was recorded at the peak of the THz waveform for a series of pump–probe delays, yielding the time-dependent evolution of THz transmission following photoexcitation.

### GIWAXS Measurements

The GIWAXS measurements were conducted using a Rigaku Smartlab X-ray diffractometer with Cu–Kα X-rays as source and using a HyPix-3000 2D X-ray detector in a Bragg–Brentano reflection geometry.

## Supplementary Material



## Notes

Organic spacer cations, X-ray diffraction measurements, scanning electron microscopy, absorption spectra, transient PL via iCCD and TCSPC, simulations of excitation diffusion, details of OPTP measurements, method for charge-carrier mobility extraction and GIWAXS measurements (PDF)
