# Supplementary material for: Odd–Even Cation Engineering of the Excitation Transport Anisotropy in Two-Dimensional Perovskite Films
Source: ACS Nano. 2026 May 20;20(21):15706–15. doi: 10.1021/acsnano.6c05301 (PMC13235636; doi:10.1021/acsnano.6c05301)
Supplement: Supplementary file 1 [file nn6c05301_si_001.pdf]

Supporting Information: Odd-Even Cation Engineering of the Excitation Transport Anisotropy in Two-Dimensional Perovskite Films

Jiaxing Du[a], *Marcello Righetto*[a, b], *Maryam Choghaei*[c], *Siyu Yan*[a], *Christopher A. Wallerius*[d], *Klaus Meerholz*[d], *Michael B. Johnston*[a], *Selina Olthof*[c], *Laura M. Herz*[a, *]

[a] Department of Physics, University of Oxford, Clarendon Laboratory, Parks Road, OX1 3PU, United Kingdom

[b] Department of Chemical Science, Università degli Studi di Padova, Via Marzolo 1, 35131 Padova, Italy

[c] Wuppertal Center for Smart Materials & Systems (CM@S), University of Wuppertal, 42119 Wuppertal, Germany

[d] Department Chemistry, University of Cologne, 50939 Cologne, Germany

Content

1. Organic spacer cations....3
2. X-ray Diffraction Measurements....4
3. Scanning Electron Microscopy....7
4. Absorption spectra....12
5. Transient photoluminescence (PL) measurements....13
   5.1 Intensified charge coupled device (iCCD)....13
   5.2 Time-correlated single photon counting (TCSPC)....13
   5.3 TRPL Decay Modeling....14
6. Simulations capturing diffusion of excitations....19
   6.1 One-dimensional diffusion equation....19
   6.2 Surface recombination....19
   6.3 Expression of observed PL spectra....21
   6.4 Determination of diffusion coefficients....26
   6.5 Ruling out the influence of low-energy emission peaks....27
   6.6 Subdiffusion in measurements....30
7. Optical Pump THz Probe (OPTP) Measurements....31
   7.1 OPTP measurement details....31
   7.2 Extraction of THz Charge-Carrier Mobility....31
8. Grazing Incidence Wide Angle X-ray Scattering (GIWAXS) Measurements....38
   8.1 GIWAXS measurement details....38
   8.2 Depth-dependent GIWAXS measurements....48

References....52

1. Organic spacer cations

For this study of two-dimensional $(Cx)_2PbI_4$ perovskites, a series of alkylammonium iodide salts ($C_xH_{2x+1}NH_3I$), as listed in the main manuscript, were employed. The carbon chain length of the spacer cations ranged from x=3 (n-propylammonium iodide, labeled as C3) to x=8 (*n*-octylammonium iodide, labeled as C8).

Label	Organic spacer cation	Chemical Formula	RP 2D Perovskite Formula
C3	*n*-Propylammonium	$C_3H_7NH_3^+$	$(C_3H_7NH_3)_2PbI_4$
C4	*n*-Butylammonium	$C_4H_9NH_3^+$	$(C_4H_9NH_3)_2PbI_4$
C5	*n*-Pentylammonium	$C_5H_{11}NH_3^+$	$(C_5H_{11}NH_3)_2PbI_4$
C6	*n*-Hexylammonium	$C_6H_{13}NH_3^+$	$(C_6H_{13}NH_3)_2PbI_4$
C7	*n*-Hepthylammonium	$C_7H_{15}NH_3^+$	$(C_7H_{15}NH_3)_2PbI_4$
C8	*n*-Octylammonium	$C_8H_{17}NH_3^+$	$(C_8H_{17}NH_3)_2PbI_4$

Table S1. Summary of the organic spacer cations employed in this study and their corresponding labels, ranging from *n*-propylammonium (C3) to *n*-octylammonium (C8).

2. X-ray Diffraction Measurements

X-ray diffraction measurements (XRD) were taken using a Panalytical X-pert powder diffractometer. The Cu-Kα X-ray source (λ=1.54 Å) was set to 40 kV voltage and 40 mA current.

Our XRD data further support the conclusion that the films are dominated by the expected RP phase. All diffraction patterns are consistent with layered RP-type ordering, and no additional diffraction features are observed that would provide direct evidence for distinct non-RP phases within the resolution of the present measurements. This interpretation is also consistent with the PL and absorption spectra shown in the main text, which display the characteristic optical response expected for RP-phase lead-iodide 2D perovskites.

To further examine the structural evolution across the alkyl spacer series, we analyzed the full-width-at-half-maximum of the (002) diffraction peaks in the XRD patterns of the C3–C8 films (Figure S2 (A)). The extracted FWHM values show that the (002) peak becomes broader for films containing longer alkyl spacer cations (Figure S2 (B)), indicating an increasing contribution from structural disorder with chain length.

This trend is consistent with the top-view SEM images (Figure S9), which suggest that the higher-carbon-number films gradually become slightly less homogeneous, and is also in line with our previous report on related lead-iodide 2DP thin films[1].

At the same time, the XRD peak broadening mainly reflects a chain-length-dependent increase in structural disorder rather than a clear odd−even alternation. The more direct parity-dependent structural difference is instead revealed by the GIWAXS measurements discussed in the main text. Together, these results suggest that the pronounced odd–even behavior in the optical and transport properties is more directly related to parity-dependent nanostructural organization, while the XRD broadening and SEM observations reflect an additional structural effect occurring gradually with increasing spacer length.

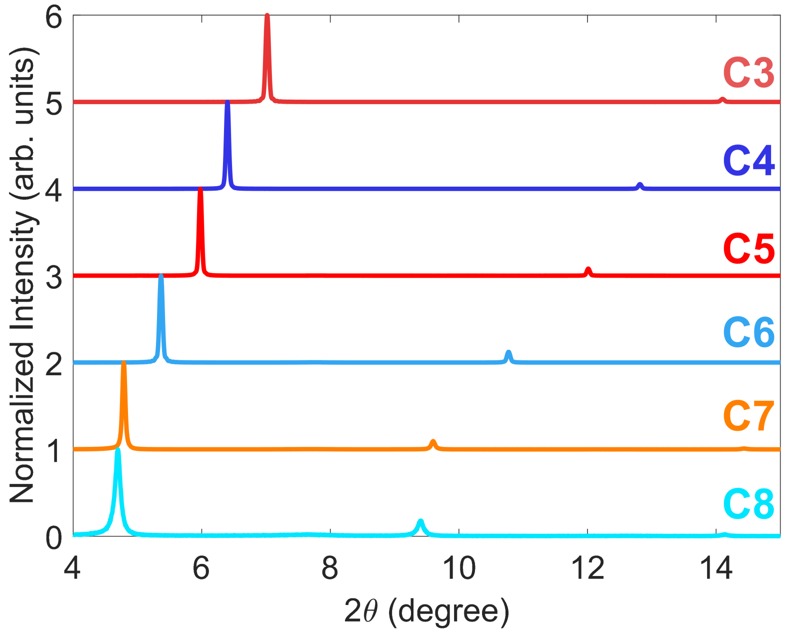

Figure S1. XRD patterns of $(Cx)_2PbI_4$ n=1 Ruddlesden–Popper 2DPs incorporating a series of non-conjugated alkyl spacer cations, ranging from n-propylammonium (C3) to n-octylammonium (C8).

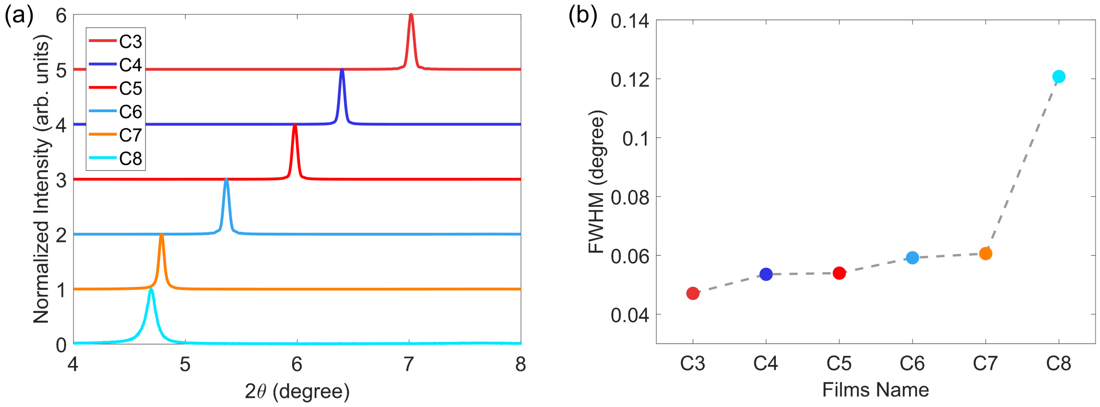

Figure S2 (a) Zoomed-in region of XRD patterns and (b) Full-width-at-half-maximum (FWHM) of (002) XRD diffraction peaks for $(Cx)_2PbI_4$ n=1 Ruddlesden–Popper 2DP films incorporating a series of non-conjugated alkyl spacer cations, ranging from n-propylammonium (C3) to n-octylammonium (C8).

3. Scanning Electron Microscopy

The thicknesses of the perovskite films were determined from cross-sectional scanning electron microscopy (SEM) images. The images were taken by a Hitachi S-4300 microscope utilizing the secondary electron detection mode, with 5 kV accelerating voltage and 10 μA emission current. The thickness of the films then derived from the cross-section SEM images as shown in the figures below.

To examine whether surface morphology could contribute to the observed odd–even variation in the optical and transport properties, we further analyzed top-view SEM images recorded for films across the C3–C8 series (Figure S9). Overall, the films show broadly similar surface morphologies, without a clear or systematic odd–even alternation in grain appearance, surface coverage, or lateral structural features. This suggests that surface morphology alone is unlikely to account for the pronounced odd–even dependence observed in the PL lifetimes and measurements of excitation transport. Furthermore, we note that the active area probed in the optical measurements is substantially larger than that of the typical grain size. The reported optical data therefore reflects an average response from many grains, rather than a local signal dominated by a particular grain or domain. This strongly reduces the likelihood that the observed trends arise from local morphological fluctuations. Instead, the odd–even effect is more consistently correlated with the parity-dependent nanostructural organization revealed by GIWAXS (Figure 4 in main text).

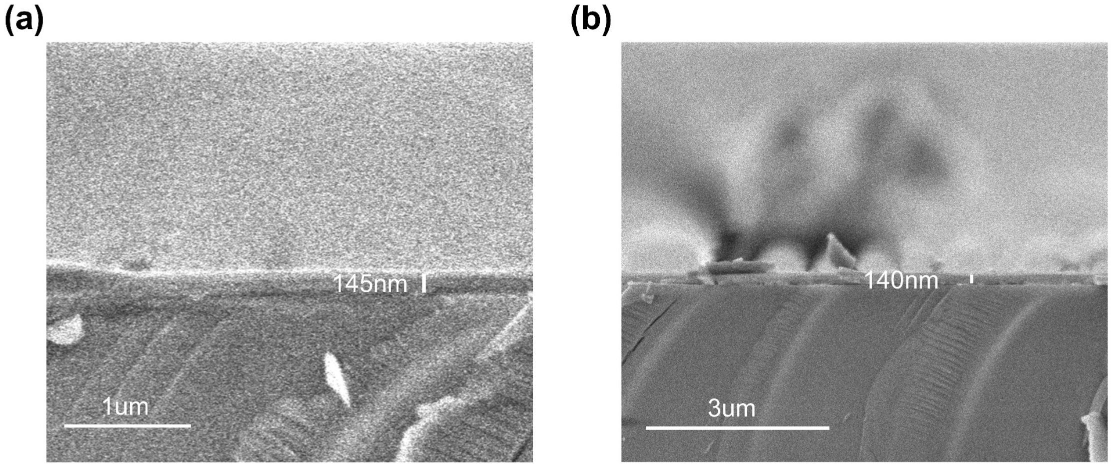

Figure S3. (a) and (b) Cross-section SEM of $(C3)_2PbI_4$ n=1 Ruddlesden–Popper 2DPs film with n-propylammonium (C3) organic spacer cation.

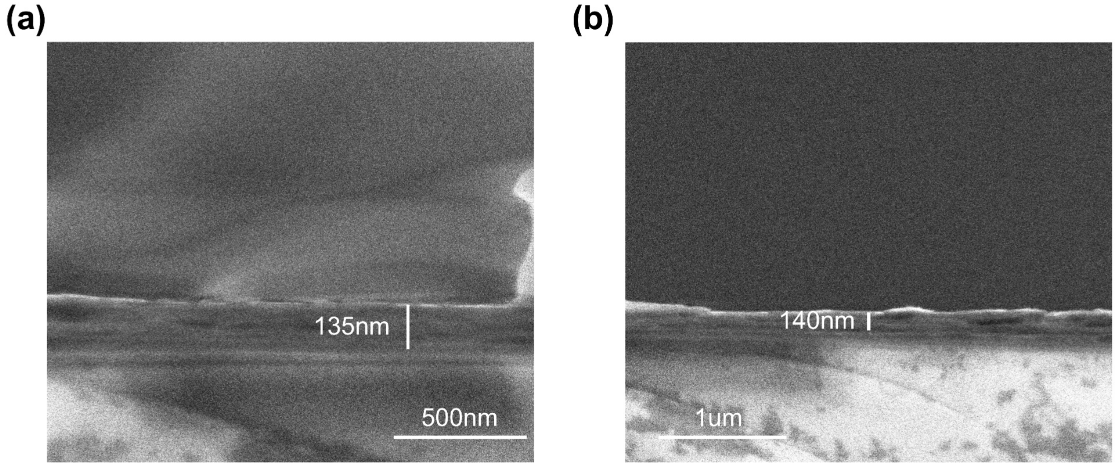

Figure S4. (a) and (b) Cross-section SEM of $(C4)_2PbI_4$ n=1 Ruddlesden–Popper 2DPs film with n-Butylammonium (C4) organic spacer cation.

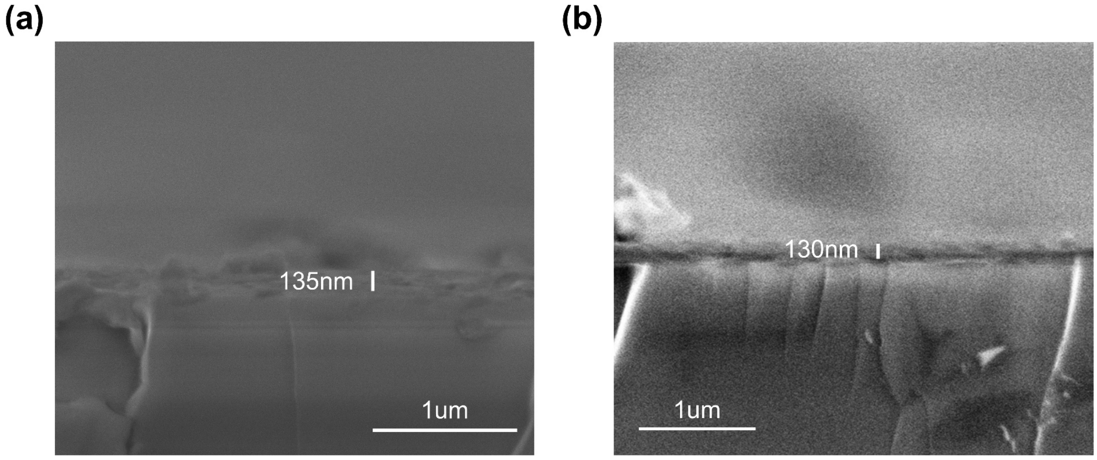

Figure S5. (a) and (b) Cross-section SEM of $(C5)_2PbI_4$ n=1 Ruddlesden–Popper 2DPs film with n-Pentylammonium (C5) organic spacer cation.

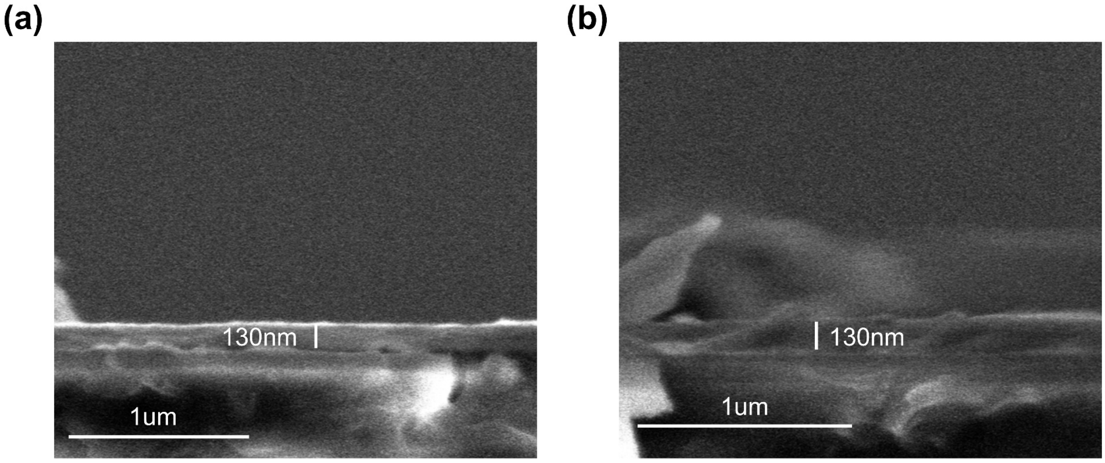

Figure S6. (a) and (b) Cross-section SEM of $(C6)_2PbI_4$ n=1 Ruddlesden–Popper 2DPs film with n-Hexylammonium (C6) organic spacer cation.

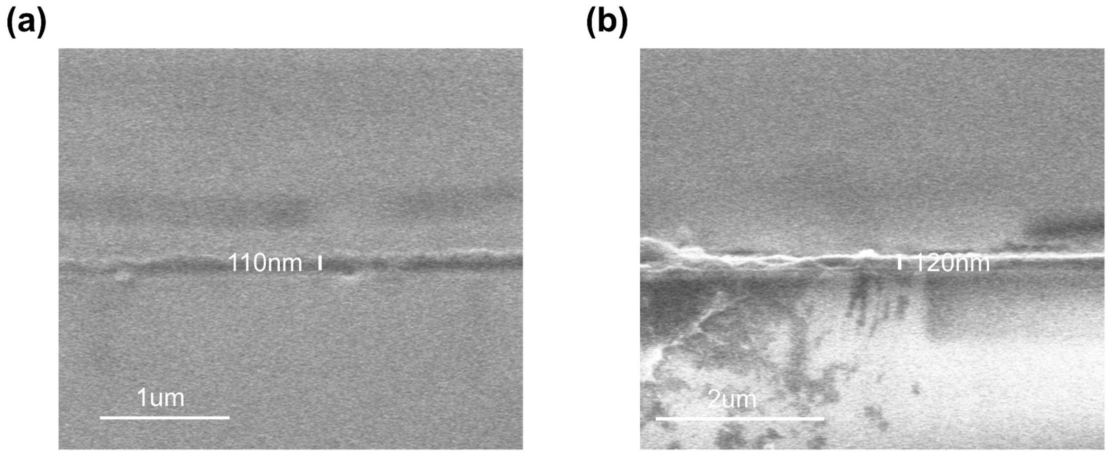

Figure S7. (a) and (b) Cross-section SEM of $(C7)_2PbI_4$ n=1 Ruddlesden–Popper 2DPs film with n-Hepthylammonium (C7) organic spacer cation.

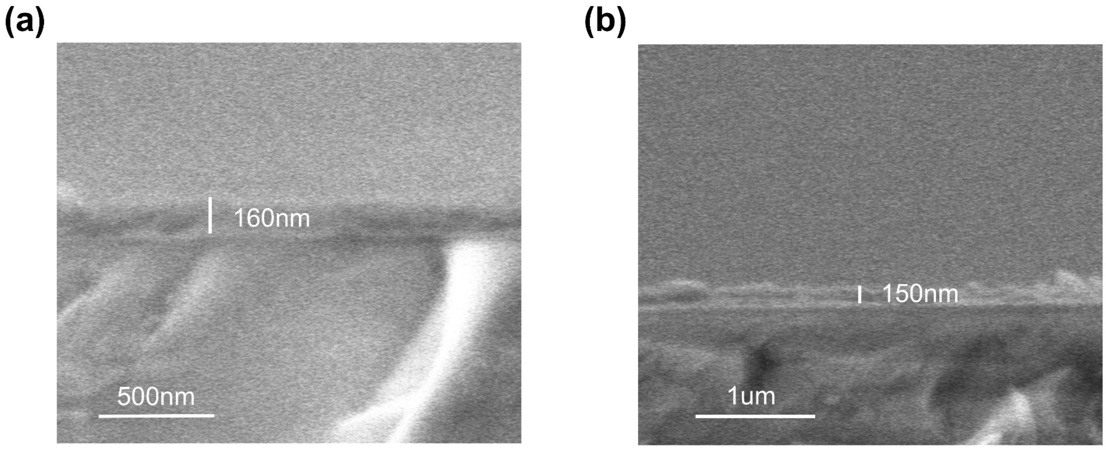

Figure S8. (a) and (b) Cross-section SEM of $(C8)_2PbI_4$ n=1 Ruddlesden–Popper 2DPs film with n-Octylammonium (C8) organic spacer cation.

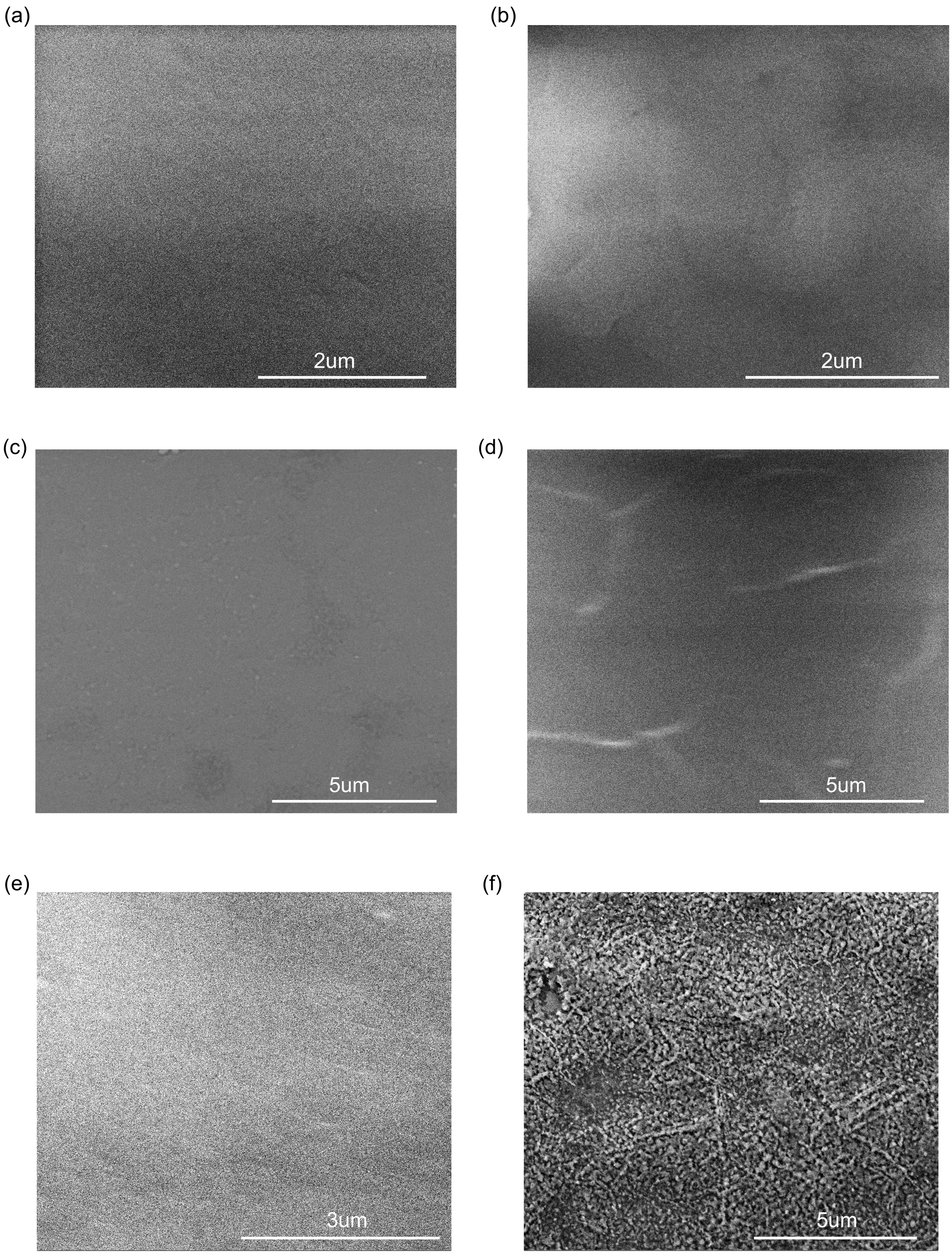

Figure S9 Top-view SEM images of RP lead-iodide 2D perovskite thin films with organic spacers from (A) C3 through to (F) C8.

4. Absorption spectra

Absorption spectra were collected using a Fourier-transform infrared (FTIR) spectrometer (Bruker Vertex 80v) equipped with a xenon lamp source, a calcium fluoride beam splitter, and a silicon diode detector. A silver mirror and z-cut quartz substrates were used as references for reflection and transmission measurements, respectively. The absorption coefficient (α) was calculated using the following equation:

$$\alpha = \frac{-\ln\left(\frac{T}{1-R}\right)}{D} \tag{1}$$

where T is the transmittance, R is the reflectance and *D* is the film thickness.

5. Transient photoluminescence (PL) measurements

5.1 Intensified charge coupled device (iCCD)

Time-resolved photoluminescence measurements were performed using a gated intensified charge-coupled device (iCCD) to record PL spectra at defined time delays following photoexcitation. A 398 nm diode laser (PicoQuant LDH-D-C-398M) with a repetition rate of 1 MHz and a fluence of 115 nJ cm^{-2} was used to excite the perovskite thin films. The emitted PL was dispersed by a grating spectrometer (Princeton Instruments SP-2558) and subsequently detected by a silicon iCCD detector (PI-MAX4, Princeton Instruments). The excitation source and detection system were synchronized using a Keysight Technologies 33600A Trueform waveform generator. All PL measurements were conducted under vacuum conditions (~3×10^{-2} mbar) by placing the samples inside a sealed vacuum chamber.

It is important to note that the spectra recorded at long delay times remain of sufficiently high quality for quantitative analysis because of the strong amplification provided by the intensified CCD. In particular, even the spectra collected at 60 ns retain good signal-to-noise ratio and spectral resolution. Using the definition

$$\mathrm{SNR} = \frac{I_{\mathrm{peak}} - \bar{I}_{\mathrm{noise}}}{\sigma_{\mathrm{noise}}}$$

the 60 ns spectrum of C3 yields an SNR of approximately 610, confirming that the late-time data should not be regarded as substantially less reliable than the earlier-time spectra for the purposes of the present analysis.

5.2 Time-correlated single photon counting (TCSPC)

For the TRPL transients shown in Figure 1D of main manuscript, the TCSPC technique was used to record the data, employing a 398 nm picosecond pulsed diode laser with a 1 MHz repetition rate for excitation. A PicoHarp 300 TCSPC event timer was used to synchronize and control the timing of photon detection. The emitted photons were detected using a single-photon avalanche photodiode (PDM series, MPD).

5.3 TRPL Decay Modeling

We analyzed the TCSPC-recorded TRPL dynamics of n = 1 Ruddlesden–Popper $(Cx)_2PbI_4$ 2D perovskites incorporating a series of non-conjugated alkyl spacer cations, ranging from n-propylammonium (C3) to n-octylammonium (C8). The PL transients displayed in Figure 1d of the main manuscript exhibit slightly dispersive decay dynamics, with an initial fast decay that slows gradually over time to exhibit longer-lived components that enable accurate recording of PL spectra with the highly sensitive iCCD camera over the first tens of nanoseconds. To capture such decay phenomenologically and extract a value for a decay time, the PL decay curves were fitted using a biexponential decay function:

$$I(t) = A_1 e^{-t/\tau_1} + A_2 e^{-t/\tau_2},$$

where A_1 and A_2 are the amplitudes of the fast and slow decay components, and τ_1 and τ_2 are the corresponding lifetimes. The average lifetime was calculated as:

$$\langle\tau\rangle = \frac{A_1\tau_1 + A_2\tau_2}{A_1 + A_2}.$$

Extracted values are shown in Table S2 below. We note that these values mostly reflect the initial 1/e decay of the PL transients which is likely to be strongly affected by the density of defects accumulating at grain boundaries, and therefore is related to the degree of crystalline ordering.

Label (odd)	Average Lifetime (ns)	Label (even)	Average Lifetime (ns)
C3	0.33	C4	0.62
C5	0.31	C6	0.62
C7	0.32	C8	0.61

Table S2. Summary of average lifetimes of the PL emitted from thin films of $(Cx)_2PbI_4$ n=1 Ruddlesden–Popper 2DPs incorporating a series of non-conjugated alkylammonium spacer cations, ranging from *n*-propylammonium (C3) to *n*-octylammonium (C8).

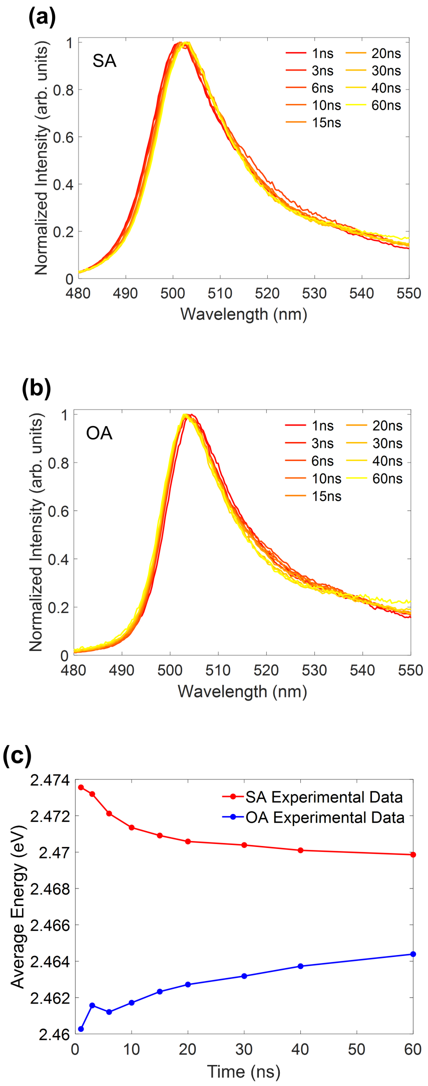

Figure S10. Charge-carrier diffusion measurement for a $(C3)_2PbI_4$ thin film, deduced from time-resolved PL spectra recorded with a gated iCCD. (a)Time-dependent PL spectra recorded in same-side collection, air-side excitation (SA) configuration and (b) Time-dependent PL spectra recorded in opposite-side collection, air-side excitation (OA) configuration, for a range of times up to 60ns after excitation. (c) Average energy values extracted for OA and SA configurations as a function of time after excitation.

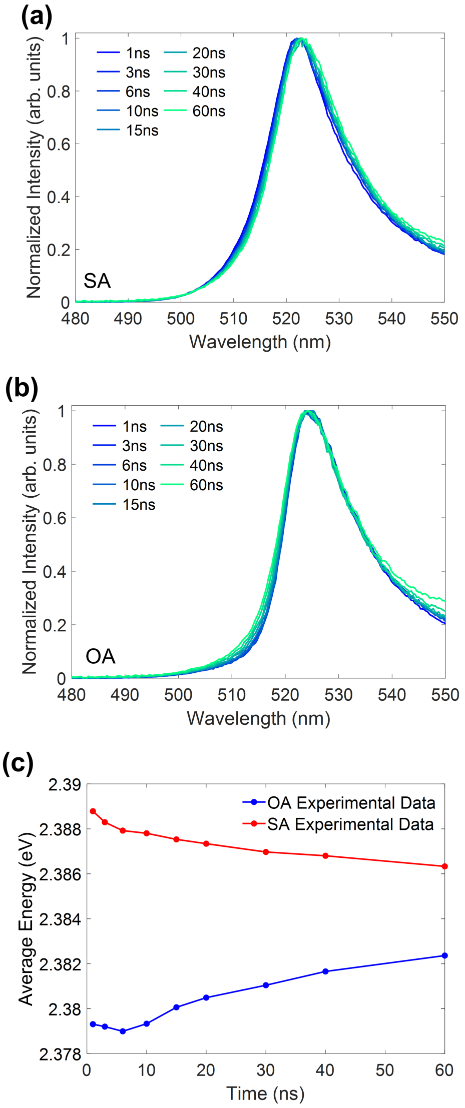

Figure S11. Charge-carrier diffusion measurement for a $(C6)_2PbI_4$ thin film, deduced from time-resolved PL spectra recorded with a gated iCCD. (a)Time-dependent PL spectra recorded in SA configuration and (b) Time-dependent PL spectra recorded in OA configuration, for a range of times up to 60ns after excitation. (c) Average energy values extracted for OA and SA configurations as a function of time after excitation.

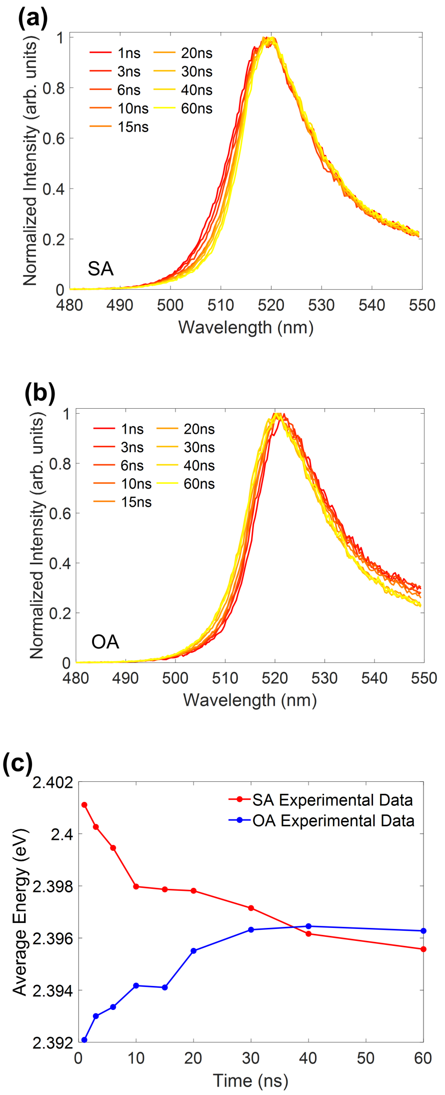

Figure S12. Charge-carrier diffusion measurement for $(C7)_2PbI_4$ thin film, deduced from time-resolved PL spectra recorded with a gated iCCD. (a)Time-dependent PL spectra recorded in SA configuration and (b) Time-dependent PL spectra recorded in OA configuration, for a range of times up to 60ns after excitation. (c) Average energy values extracted for OA and SA configurations as a function of time after excitation.

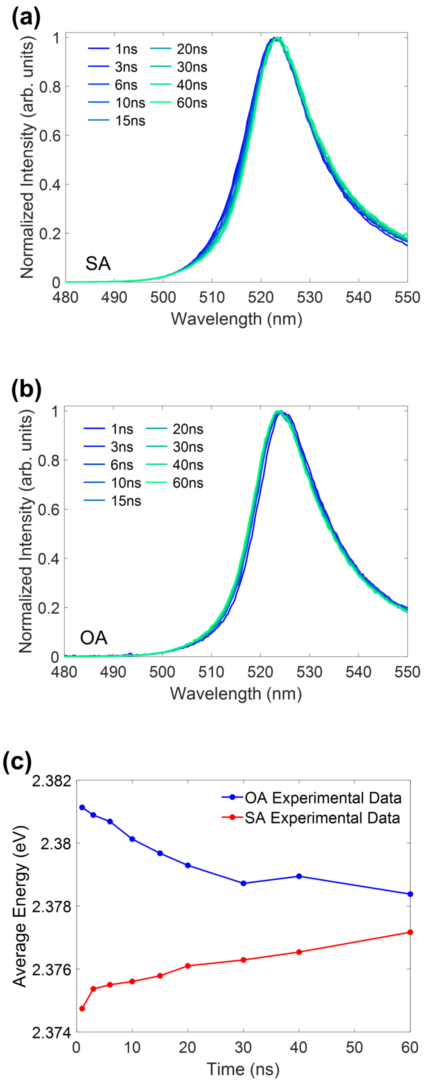

Figure S13. Charge-carrier diffusion measurement for $(C8)_2PbI_4$ thin film, deduced from time-resolved PL spectra recorded with a gated iCCD. (a)Time-dependent PL spectra recorded in SA configuration and (b) Time-dependent PL spectra recorded in OA configuration, for a range of times up to 60ns after excitation. (c) Average energy values extracted for OA and SA configurations as a function of time after excitation.

6. Simulations capturing diffusion of excitations

6.1 One-dimensional diffusion equation

To simulate charge-carrier diffusion in perovskite thin films, we employed a one-dimensional (1D) diffusion model, since the lateral excitation spot size (~400 μm) is much larger than the film thickness. Under this condition, a significant excitation density gradient $n(x,t)$ exists only along the direction x normal to the film surface (i.e., along the depth profile). The 1D diffusion equation is expressed as[2]:

$$\frac{\partial n(x,t)}{\partial t} = D\frac{\partial^2 n(x,t)}{\partial x^2} - k_1 n(x,t) \quad (2)$$

where $n(x,t)$ is excitation density, D is the diffusion coefficient, k_1 is the monomolecular recombination rate. Higher-order recombination terms were neglected as a first-order approximation, as the shape of the PL transients was found to be independent of the excitation fluence.

6.2 Surface recombination

The surface recombination near the interface between the perovskite layer and air in the simulations was determined based on our previous work[2]. The boundary conditions for the thin film were defined as follows:

$$\left.\frac{\partial n(x,t)}{\partial x}\right|_{\text{Air}} = \frac{S}{D}n(x,t) \qquad \left.\frac{\partial n(x,t)}{\partial x}\right|_{\text{Quartz}} = 0$$

where S is the surface recombination velocity.

The value of S can be derived from the following relation:

$$\frac{1}{\tau_e} = \frac{1}{\tau_b} + \frac{1}{\tau_s} \quad (3)$$

where the surface lifetime (τ_s) of excitations is determined by the effective lifetime (τ_e) which is obtained from the results in main manuscript (Figure 1D) and a bulk lifetime (τ_b).

For a semiconductor film of thickness L exhibiting a large value of S for air-side (defective) surface, and negligible S on the other (high-quality) surface, τ_s can be approximated as[3]:

$$\tau_s = \frac{L}{S} + \frac{4}{D}\left(\frac{L}{\pi}\right)^2 \quad (4)$$

Since the second term (i.e., the diffusion contribution) in Eq. (4) is much smaller than the first term (see calculations below), the surface lifetime is primarily determined by the first term. Therefore, Eq. (3) above can be approximated as follows:

$$\frac{1}{\tau_e} = \frac{1}{\tau_b} + \frac{S}{L} \tag{5}$$

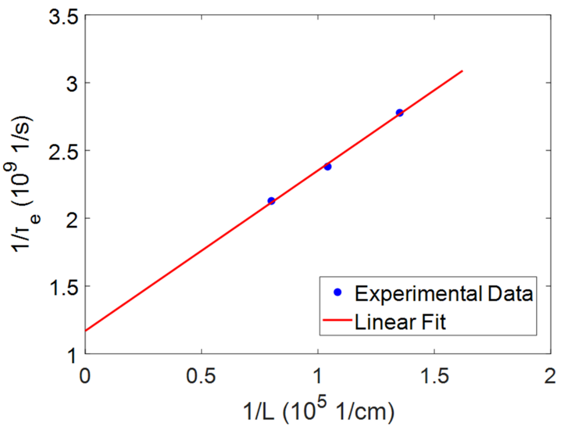

Figure S14. Extraction of a value for the surface recombination velocity S for a 2DPs $(C3)_2PbI_4$ thin film from linear fits to the effective recombination rate versus the inverse film thickness.

6.3 Expression of observed PL spectra

To enable a direct comparison between the simulation results and the experimentally measured PL spectra, it is necessary to convert the excitation density $n(x, t_1)$ at a specific time delay t_1 into the corresponding photoluminescence spectrum $I_{\mathrm{PL}}(\lambda, t_1)$ at that moment. The observed PL spectrum at a given time, which includes the effects of photon reabsorption, can be expressed as[4]:

$$I_{PL}(\lambda, t) \propto \int_0^L I_{IPL}(\lambda) e^{-\alpha(\lambda)x} n^2(x, t) dx \tag{6}$$

Here, L is the thickness of the film, $I_{\mathrm{IPL}}(\lambda)$ represents the intrinsic PL spectrum, and $\alpha(\lambda)$ is the absorption coefficient. The intrinsic PL spectrum is calculated based on Beer's law and derived from the PL spectrum recorded within the first nanosecond after excitation using the iCCD technique.

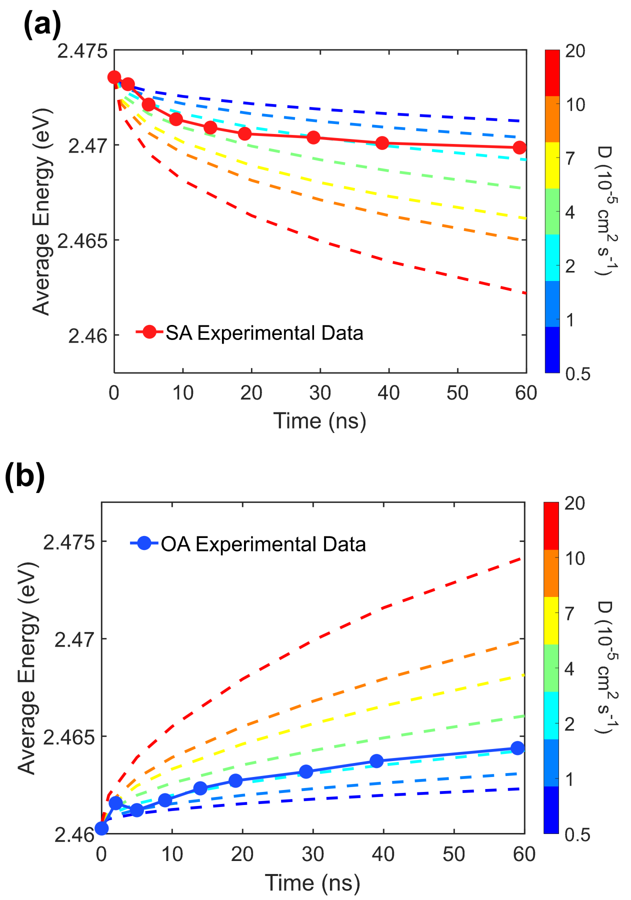

Figure S15. Simulations of excitation diffusion and determination of time-dependent diffusion coefficients for the experimental SA and OA configurations for a $(C3)_2PbI_4$ 2DPs thin film. (a) and (b) Comparison between experimentally derived values of the average energy of detected PL photons (SA: red dots; OA: blue dots) and those derived from simulations based on diffusion coefficients ranging from 0.5×10^{-5} to 20×10^{-5} cm^2/s.

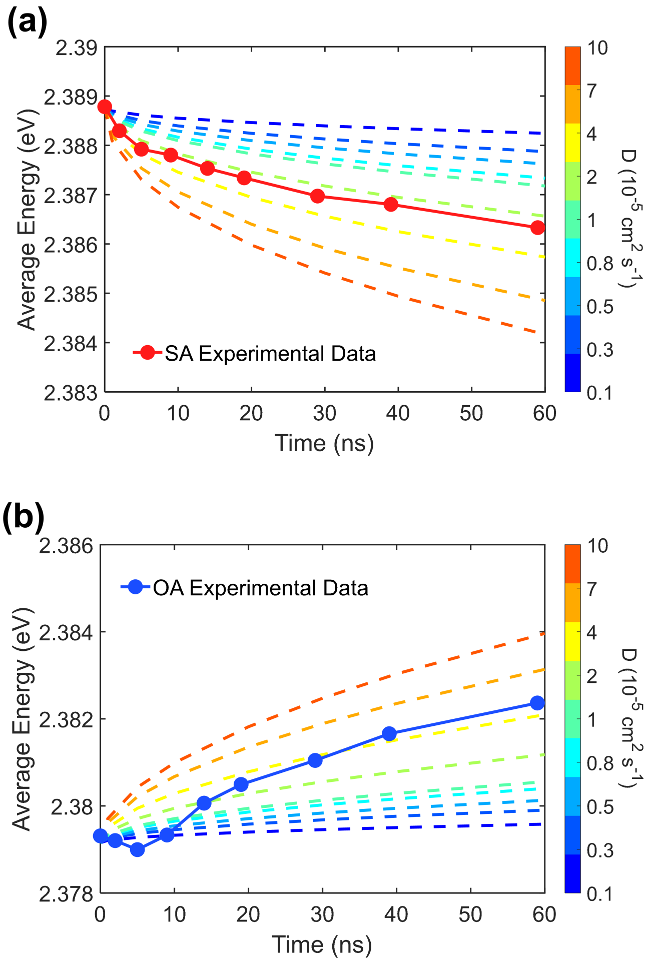

Figure S16. Simulations of excitation diffusion and determination of time-dependent diffusion coefficients for the experimental SA and OA configurations for a $(C6)_2PbI_4$ 2DPs thin film. (a) and (b) Comparison between experimentally derived values of the average energy of detected PL photons (SA: red dots; OA: blue dots) and those derived from simulations based on diffusion coefficients ranging from 0.1×10^{-5} to 10×10^{-5} cm^2/s.

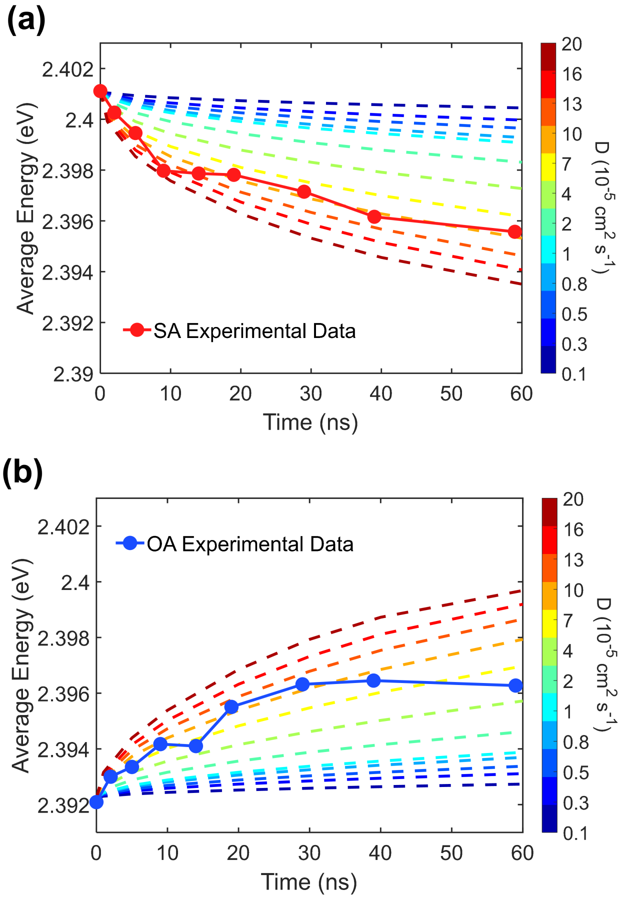

Figure S17. Simulations of excitation diffusion and determination of time-dependent diffusion coefficients for the experimental SA and OA configurations for a $(C7)_2PbI_4$ 2DPs thin film. (a) and (b) Comparison between experimentally derived values of the average energy of detected PL photons (SA: red dots; OA: blue dots) and those derived from simulations based on diffusion coefficients ranging from 0.1×10^{-5} to 20×10^{-5} cm^2/s.

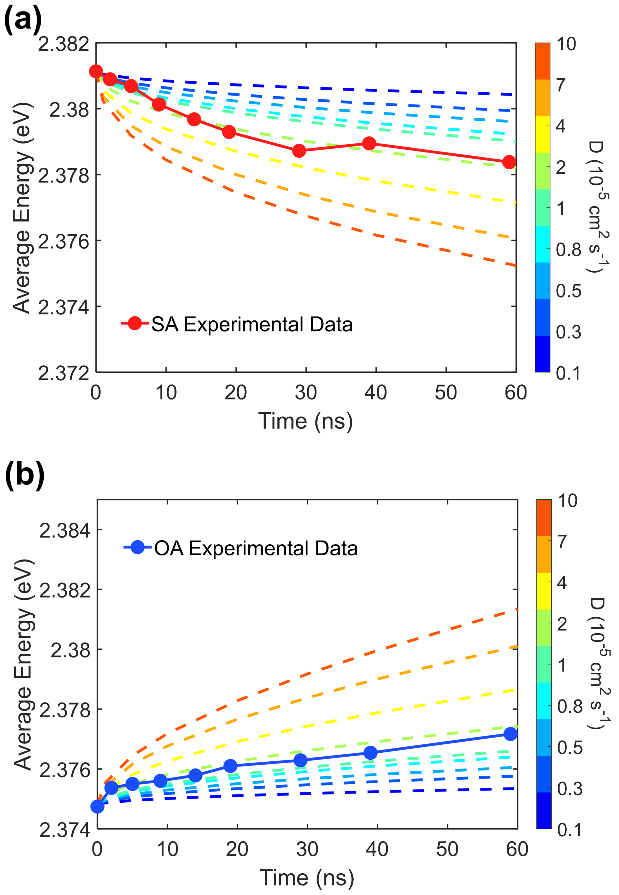

Figure S18. Simulations of excitation diffusion and determination of time-dependent diffusion coefficients for the experimental SA and OA configurations for a $(C8)_2PbI_4$ 2DPs thin film. (a) and (b) Comparison between experimentally derived values of the average energy of detected PL photons (SA: red dots; OA: blue dots) and those derived from simulations based on diffusion coefficients ranging from 0.1×10^{-5} to 10×10^{-5} cm^2/s.

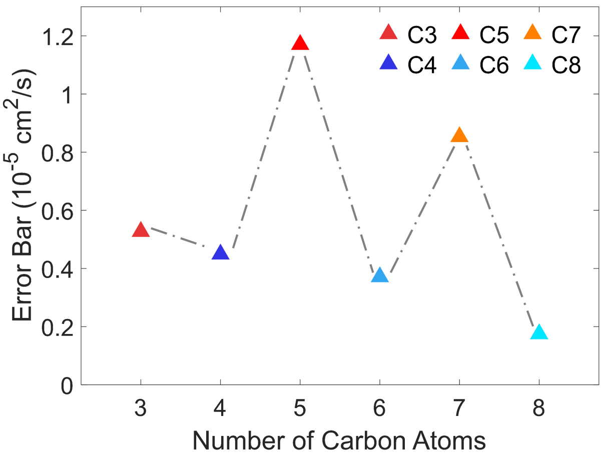

Figure S19. The magnitude of the standard errors for the mean value extracted for the diffusion coefficient from comparison of the experimental data and simulations for $(Cx)_2PbI_4$ RP 2DP films incorporating a series of non-conjugated alkylammonium spacer cations, ranging from C3 to C8.

6.4 Determination of diffusion coefficients.

To derive the excitation diffusion coefficients for the 2DP thin films, the experimentally measured variations in average PL energy were compared with simulated results obtained for different diffusion coefficients, as shown in Figures S15–S18.

For each delay time, the experimental average-energy shift was compared with the simulated result to determine the corresponding diffusion coefficient D. The diffusion coefficient reported for each film is therefore not extracted from a single time point but obtained as the mean value of the time-dependent D values derived over the full measured temporal range. This procedure reduces the influence of any individual data point, particularly at longer delay times where the signal intensity is lower and provides a more robust estimate of the overall out-of-plane diffusion coefficient relevant to the experiment.

The corresponding deviation of the extracted time-dependent D values from the mean is reflected in the error bars of the mean diffusion coefficients. In this work, these error bars are not only an estimate of the uncertainty in the extraction procedure but also provide useful information on the degree of nanostructural disorder and the extent to which additional in-plane contributions may affect the measured out-of-plane transport response.

6.5 Ruling out the influence of low-energy emission peaks

We note that low-energy defect emission can contribute to time-dependent PL redshifts in 2D perovskites, which has been widely reported and discussed and has commonly been attributed either to a trap state arising from local compositional variations or precursor depletion during film growth, or alternatively to self-trapped states[5, 6]. Because these low-energy emission features often have different emission wavelengths and PL lifetimes to those of the main PL peak measured in our experiments, they could in principle influence diffusion coefficients extracted from the photon reabsorption method.

In the present work, we indeed observe the presence of low-energy emission in the time-dependent transient PL spectra. For example, this can be observed in Figure 2(a) and (b) of the main manuscript for the $(C4)_2PbI_4$ and $(C5)_2PbI_4$ films, which shows a low-energy tail in the PL spectrum at longer times after excitation. Similar features can also be observed in time-dependent PL spectra of the other 2DPs shown in Figure S10 - S13 of the SI. The spectral position and characteristics of this low-energy emission are consistent with previously reported trap-state emission associated with local compositional variations or precursor depletion during film growth[5]. Because the PL lifetime of these trap-related states is longer than that of the initially generated photoexcitations (free charge carriers or excitons) their contribution to the low-energy shoulder becomes increasingly pronounced at longer time delay. As a result, if one were to simply integrate the entire PL spectrum and calculate the average energy of the PL spectrum using a reabsorption-based method, the extracted values would be influenced by this low-energy emission and would show an artificially enhanced red shift. Based on the fitting approach developed by the Prins group[7], we here performed a deconvolution of the high-energy and low-energy emission components (Figure S20 in SI). This approach allows the influence of the low-energy emission on the spectral evolution of the high-energy emission to be observed more directly. Here, the PL spectrum of the $(C3)_2PbI_4$ film at 60 ns was fitted with two Voigt distributions to highlight the influence from low-energy emission.

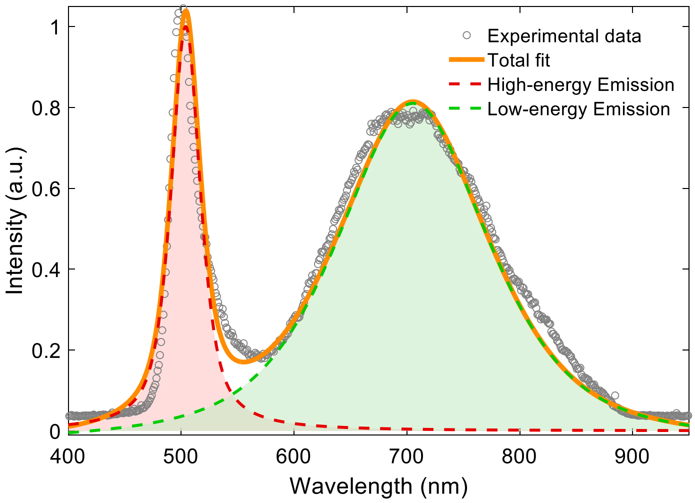

Figure S20. Fits to the C3 PL spectrum of a $(C3)_2PbI_4$ film recorded at 60 ns after excitation, based on two Voigt distributions to deconvolute the high-energy and low-energy emission.

To ensure that our analysis was unaffected by the emission from such low-energy traps, we carried out

the following four measures:

(i) We limited the spectra range included when calculating the PL average energy in order to avoid the influence of the low-energy emission described above. We selected a spectral window sufficiently far from the low-energy tail for the average-energy calculation, as mentioned in our previous article describing our method in more detail[2]. For example, for the $(C3)_2PbI_4$ film, we chose the wavelength range from 460 to 510 nm to calculate the average energy. This greatly suppresses the contribution of the low-energy emission to the extracted spectral shift.

(ii) We analysed the PL spectra only at times after excitation (<60ns) for which the free-charge and exciton response dominates the PL spectra, in order to limit the contribution from the low-energy emission (see PL spectra in SI Figures S10 – S13).

(iii) We ensured that such procedures indeed resulted in dynamics that are dominated almost exclusively by photon reabsorption effects by ascertaining that when the excitation-emission geometry is changed, the perceived red shift turns into a symmetric blue shift. For example, we measure and compare SA and OA configurations, where the former, same-side detection with respect to the excitation side, should result in a redshift of PL over time, while the latter, opposite-side detection, should result in a blue shift. If, on the other hand, low-energy emission from traps were to cause the red-shift, both geometries should show a redshift. SI Figure S21 (A) shows that indeed, if the spectra region analysed includes that associated with the defect emission, a red shift is observed for both geometries. However, Figure S21 (B) shows that if the spectra region is limited to that associated with only free charges or excitons, symmetric red- and blue-shifts are observed for the two geometries, thus proving that the dynamics originate solely from photon reabsorption effects. More importantly, if the average-energy curves obtained with the selected spectral region were still affected by the low-energy emission, they would not be expected to stabilize at a fixed average-energy value after the carrier-density profile becomes spatially flat. Instead, they should continue to exhibit a universal red shift at longer delay times, which is not what we observe in Figure S21 (B).

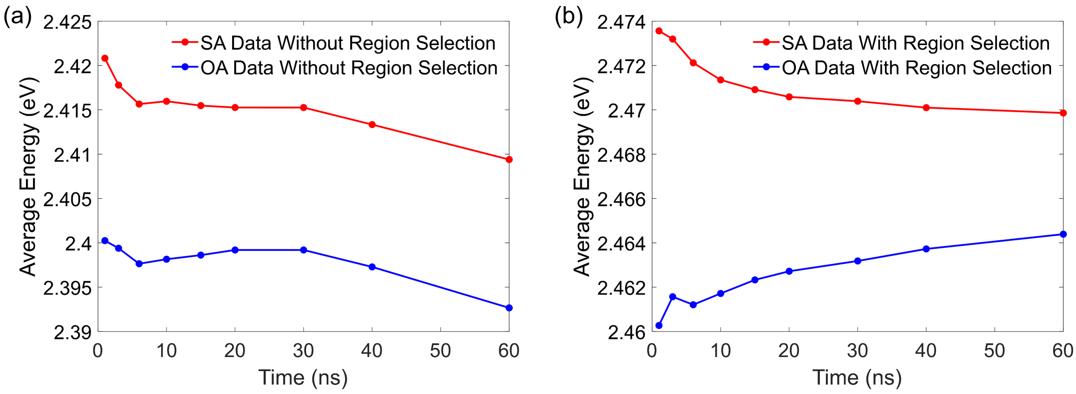

Figure S21 Average energy curves calculated for a $(C3)_2PbI_4$ film for both SA and OA configurations for the case of (a) no limitation to the PL spectral range included (460 - 550 nm) and thus including emission from a low-energy defect state, and (b) with selection of a spectra region

(460 - 510nm) in which the emission from originally excited free charges and excitons dominates.

(iv) In addition, we note that for our lead-iodide 2D perovskite thin films incorporating alkyl organic spacer cations, the relatively small Stokes shift means that the low-energy emission peak is spectrally well separated from the corresponding absorption edge (Figure S22). As a result, photon reabsorption predominantly affects the high-energy emission that overlaps with the absorption spectrum and produces the corresponding red- and blue-shift behavior observed under different measurement configurations. In contrast, the low-energy emission does not significantly overlap with the absorption and is not expected to undergo a comparable red or blue shifts caused by reabsorption effect. This further reduces the possibility that the low-energy emission feature disturbs the spectral shifts derived in our analysis.

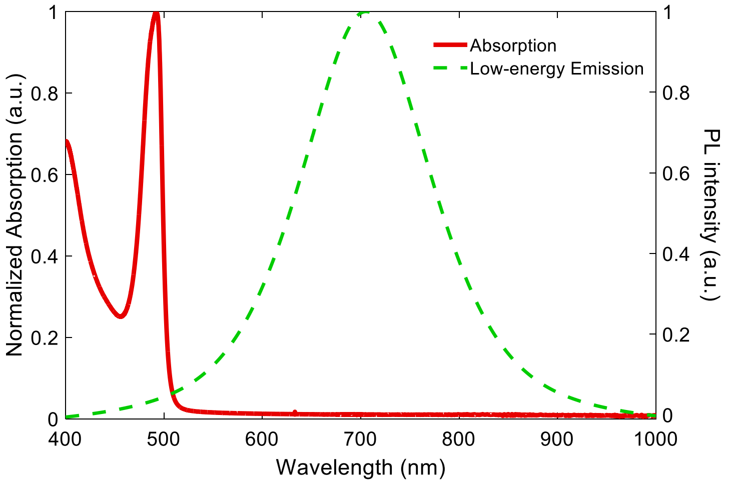

Figure S22 Comparison of absorption spectrum of $(C3)_2PbI_4$ thin films and low-energy emission extracted from PL spectrum at 60 ns time delay.

Based on the discussion and analysis above, we show that once an appropriate spectral window is selected for the average-energy calculation, the influence of the low-energy emission on our measurements becomes mostly negligible. In this situation, the extracted spectral shifts are dominated by the free-charge and excitonic emission component relevant to the photon reabsorption process of the initially generated intrinsic photoexcitations.

More broadly, reabsorption-based methods have been widely applied in recent years to probe out-of-plane diffusion in perovskite thin films[2, 8-10]. When such methods are combined with highly sensitive detection systems, such as intensified CCD cameras, the precision and sensitivity of the measurements can be significantly improved[2, 9, 10]. This makes it possible to resolve very small out-of-plane diffusion processes that were difficult to quantify accurately in the past.

6.6 Subdiffusion in measurements

To further evaluate whether sub diffusive behavior may affect the present analysis, we examined the time-dependent diffusion coefficient values extracted at each delay time from the comparison between experiment and simulation. If strong sub diffusion were present, the extracted diffusion coefficient would be expected to decrease systematically with increasing delay time, as reported previously for trap-influenced transport in layered perovskites[11]. However, this behavior is not observed in our data.

As a representative example, Figure S23 shows the average-energy dynamics of the C4 film together with the corresponding simulated curves, as well as the time-dependent diffusion coefficients extracted from these data. For this highly oriented film, within a fitting range from 0.000001 to 0.0002 cm^2/s (Figure S23 (A)) the extracted diffusion coefficients fluctuate around 0.00004 cm^2/s with an error bar of approximately 0.000015 cm^2/s (Figure S23 (B)), and do not show a systematic decrease with delay time. This indicates that, within the temporal window and sensitivity of the present experiment, the measured spectral evolution is adequately described by the one-dimensional diffusion equation.

The absence of clear subdiffusive behavior in the present measurements may have several possible explanations. First, the relatively strong XRD features (Figure S1), high SNR of GIWAXS data (Figure S30- S35), and SEM images (Figure S9) collectively indicate that the films are of comparatively high structural quality, which likely reduces the density of trap states relative to more disordered 2D perovskite thin films. Second, the excitation conditions used here may also contribute. To ensure sufficiently high signal-to-noise ratio at longer delay times, the films were excited at a fluence of 115 nJ cm^{-2}, under which partial filling of trap states may occur. This would reduce the effective trap density experienced by the photoexcitations during the measurement and could further suppress trap-assisted subdiffusive transport. We therefore conclude that, although subdiffusion is an important possibility in layered perovskites, it is not clearly evident in the present measurements within the temporal window studied here.

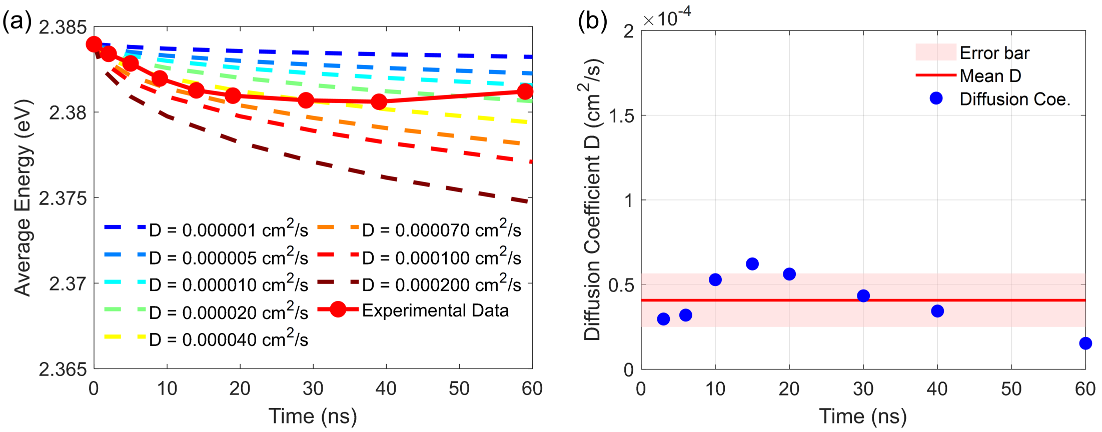

Figure S23 (a) Average photon energy (dots) and simulated results (dashed lines) for $(C4)_2PbI_4$ films for SA configuration and (b) corresponding extracted time-dependent diffusion coefficient.

7. Optical Pump THz Probe (OPTP) Measurements

7.1 OPTP measurement details.

OPTP measurements were performed using a Spectra Physics Mai Tai–Ascend–Spitfire Pro Ti:sapphire regenerative amplifier, producing 35 fs pulses at an 800 nm center wavelength and a 5 kHz repetition rate. THz probe pulses were generated using a spintronic emitter coated with antireflection and high-reflectivity layers. Samples were set in an evacuated chamber (pressure < 10^{-1} mbar) and excited by the optical pump, followed by probing with the THz pulse after a controlled delay. Pump and THz beams were modulated at 1.25 kHz and 2.5 kHz, respectively, using optical choppers to extract the pump-induced change in THz transmission (ΔT). Pump power was adjusted using a neutral-density filter wheel. THz transmission through the thin films was detected via electro-optic sampling in a 1-mm-thick (110)-oriented ZnTe crystal using a spatially and temporally overlapped 800 nm gate pulse. The THz signal was recorded at the peak of the THz waveform for a series of pump–probe delays, yielding the time-dependent evolution of THz transmission following photoexcitation.

7.2 Extraction of THz Charge-Carrier Mobility

The terahertz (THz) mobility was determined following the method described by Wehrenfennig *et al*[12]. In brief, the sheet photoconductivity ΔS of a thin film with a thickness much smaller than the THz wavelength can be expressed as:

$$\Delta S = -\varepsilon_0 c(n_a + n_b)\frac{\Delta T}{T} \tag{7}$$

where ε_0 is the vacuum permittivity, c is the speed of light, n_a and n_b are the THz refractive indices of the materials adjacent to the perovskite layer on the front and rear sides, respectively. The term $\Delta T/T$ is the ratio of the photoinduced change in the transmitted THz electric field to the transmitted field in the dark.

The initial density of photoexcited charge carriers N is given by:

$$N = \phi\frac{E\lambda}{hc}\left(1 - R_{\text{pump}} - T_{\text{pump}}\right) \tag{8}$$

where E is the incident pump pulse energy (excitation energy), λ is the excitation wavelength, ϕ is the ratio of free charges generated per absorbed photon, and R_{pump} and T_{pump} are the reflected and transmitted fractions of the pump beam, respectively.

The charge-carrier mobility μ can then be extracted from:

$$\mu = \frac{\Delta S A_{\text{eff}}}{Ne} \tag{9}$$

where $A_{\text{eff}} = 2\pi(\sigma_{\text{pump}}^2 + \sigma_{\text{THz}}^2)$ is the effective overlap area of the pump and probe beams, σ is the Gaussian beam waist and e is the elementary charge. Substituting Equations 7 and 8 into Equation 9 yields:

$$\phi\mu = -\varepsilon_0 c(n_a + n_b)\frac{hcA_{\text{eff}}}{eE\lambda(1 - R_{\text{pump}} - T_{\text{pump}})}\frac{\Delta T}{T} \tag{10}$$

From this expression, the effective charge-carrier mobility $\phi\mu$ can be determined based on the pump beam parameters and the initial measured $\Delta T/T$ of the sample. Here, μ is the electron-hole sum mobility, and ϕ is the charge-to-photon branching ratio, which is assumed to be unity at room temperature. The charge-carrier mobility for each sample was extracted by photoexciting the films at a series of different excitation fluences and performing a linear fit between E and the initial $\Delta T/T$. In our linear fitting procedure, we define the parameter A to include all terms other than E and the initial $\Delta T/T$ to simplify the fitting expression. Thus, the parameter A is given by:

$$A = \varepsilon_0 c(n_a + n_b)\frac{hcA_{\text{eff}}}{e\phi\lambda(1 - R_{\text{pump}} - T_{\text{pump}})}$$

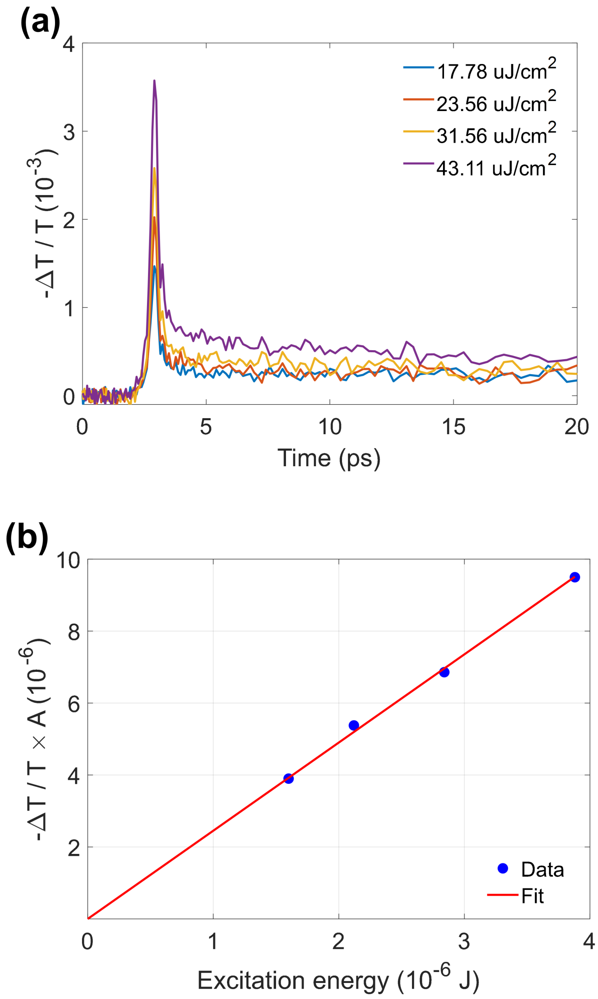

Figure S24. (a) Fluence-dependent OPTP transients for a $(C3)_2PbI_4$ thin film measured following 3.1 eV pulsed excitation with a range of different excitation fluences. (b) Linear fits between excitation energy and the initial values of $\Delta T/T$.

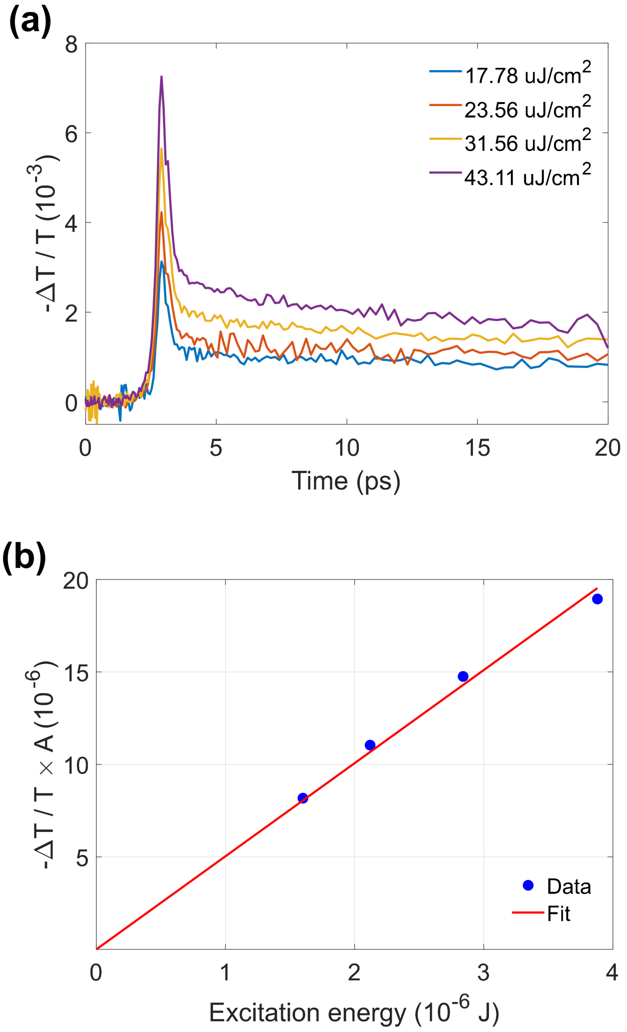

Figure S25. (a) Fluence-dependent OPTP transients for a $(C4)_2PbI_4$ thin film measured following 3.1 eV pulsed excitation with a range of different excitation fluences. (b) Linear fits between excitation energy and the initial values of $\Delta T/T$.

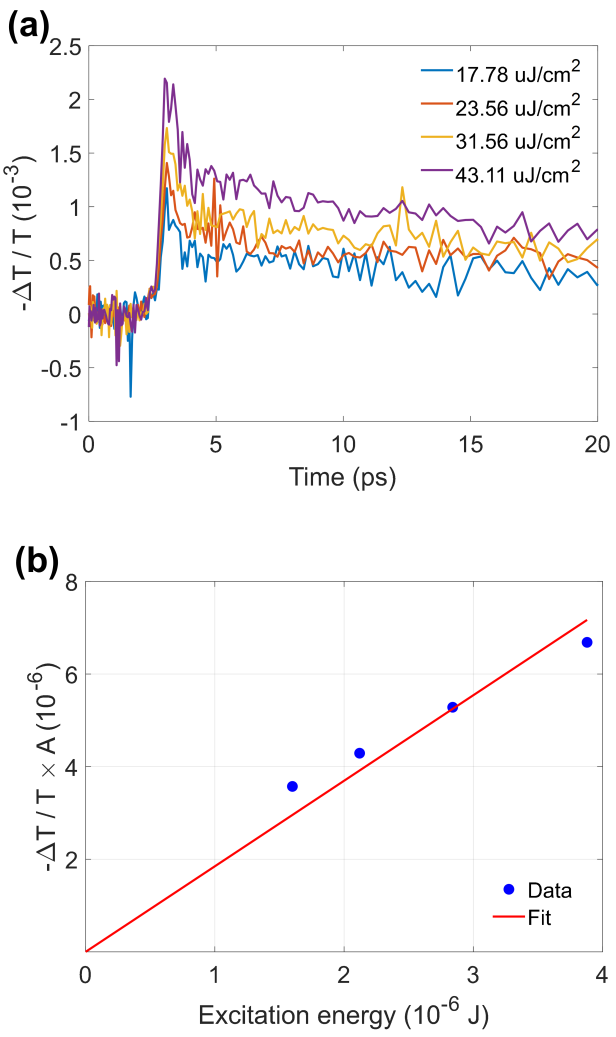

Figure S26. (a) Fluence-dependent OPTP transients for a $(C5)_2PbI_4$ thin film measured following 3.1 eV pulsed excitation with a range of different excitation fluences. (b) Linear fits between excitation energy and the initial values of $\Delta T/T$.

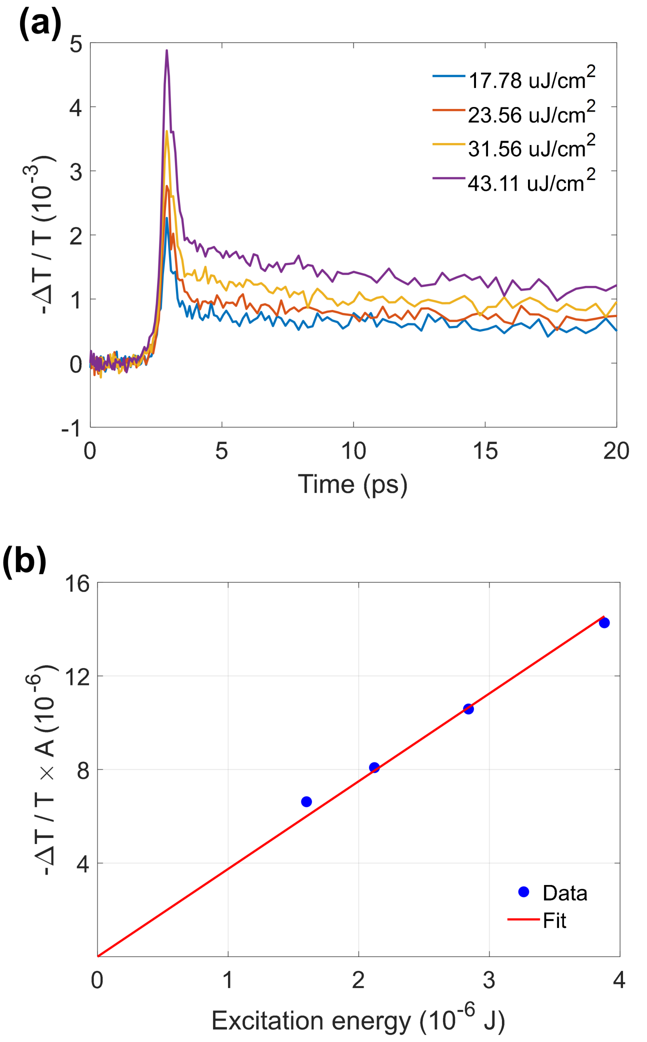

Figure S27. (a) Fluence-dependent OPTP transients for a $(C6)_2PbI_4$ thin film measured following 3.1 eV pulsed excitation with a range of different excitation fluences. (b) Linear fits between excitation energy and the initial values of $\Delta T/T$.

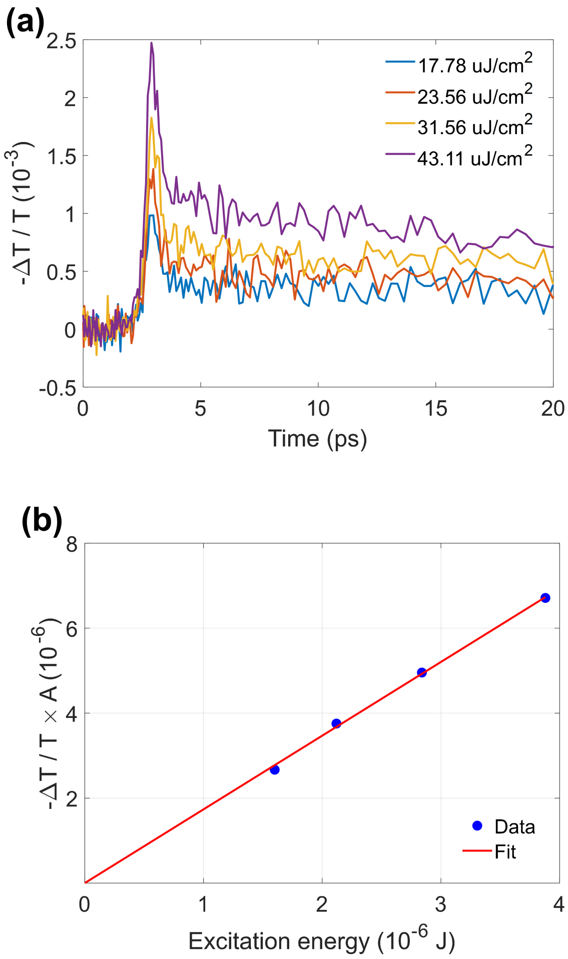

Figure S28. (a) Fluence-dependent OPTP transients for a $(C7)_2PbI_4$ thin film measured following 3.1 eV pulsed excitation with a range of different excitation fluences. (b) Linear fits between excitation energy and the initial values of $\Delta T/T$.

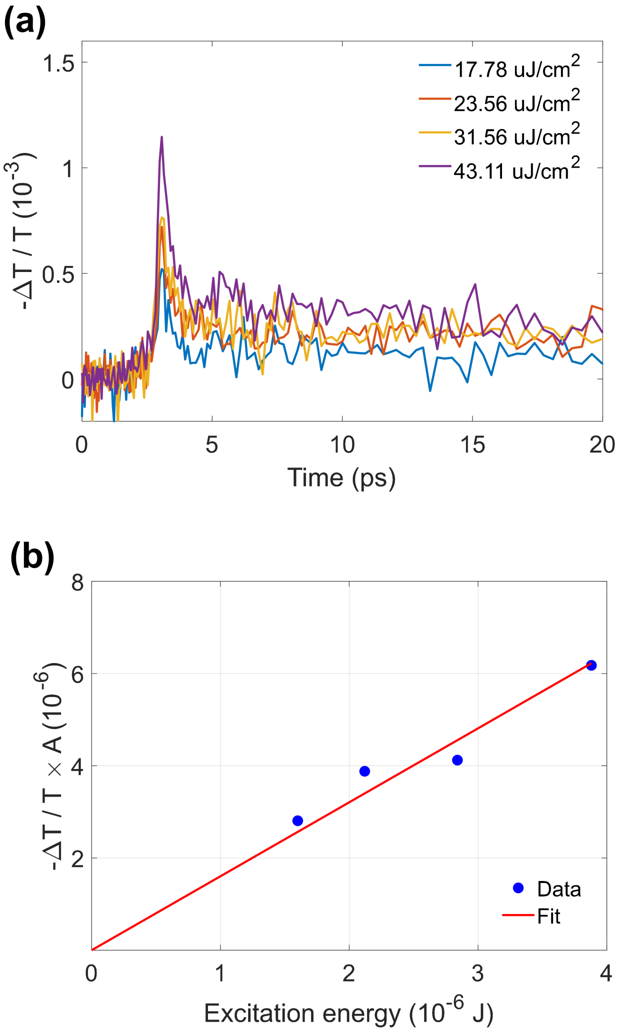

Figure S29. (a) Fluence-dependent OPTP transients for a $(C8)_2PbI_4$ thin film measured following 3.1 eV pulsed excitation with a range of different excitation fluences. (b) Linear fits between excitation energy and the initial values of $\Delta T/T$.

8. Grazing Incidence Wide Angle X-ray Scattering (GIWAXS) Measurements

8.1 GIWAXS measurement details

The GIWAXS measurements were conducted using a Rigaku Smartlab X-ray diffractometer with Cu-Kα X-rays as source and using a HyPix-3000 2D X-ray detector in a Bragg-Brentano reflection geometry. All the GIWAXS detector images were reshaped and converted to wavevector space based on the relationship between scattering vector and diffraction signal on the detector[13, 14].

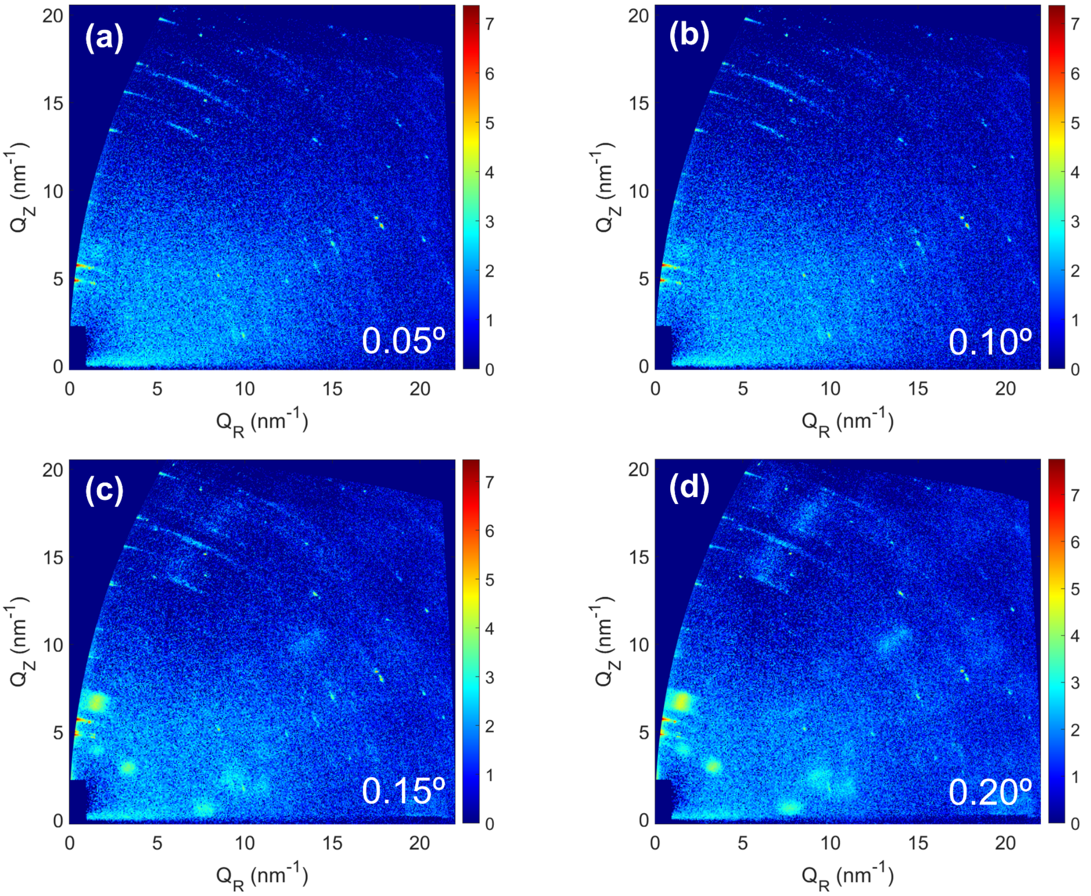

Figure S30. Depth-dependent GIWAXS images recorded for a 2DP $(C3)_2PbI_4$ thin film. The incident angle is indicated in the figures: (A) 0.05°, (B) 0.10°, (C) 0.15°, (D) 0.20°.

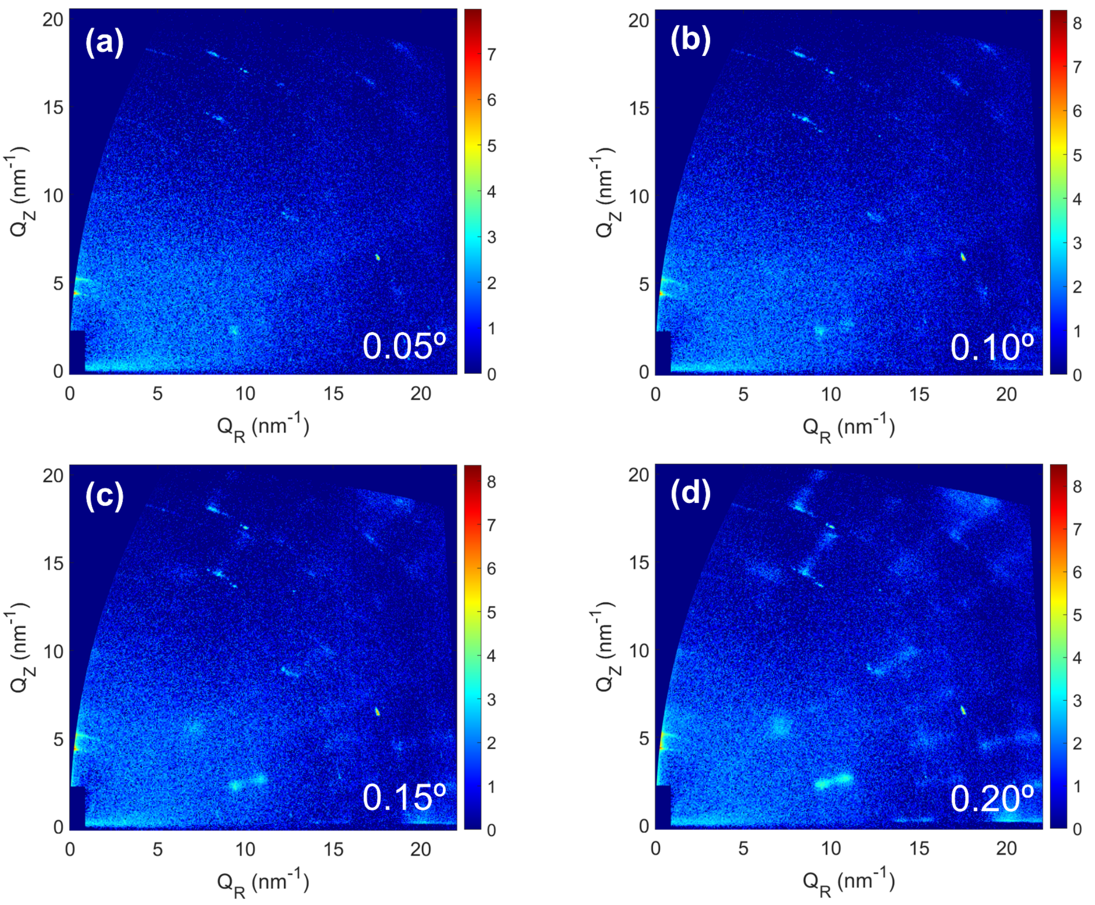

Figure S31. Depth-dependent GIWAXS images recorded for a 2DP $(C4)_2PbI_4$ thin film. The incident angle is indicated in the figures: (A) 0.05°, (B) 0.10°, (C) 0.15°, (D) 0.20°.

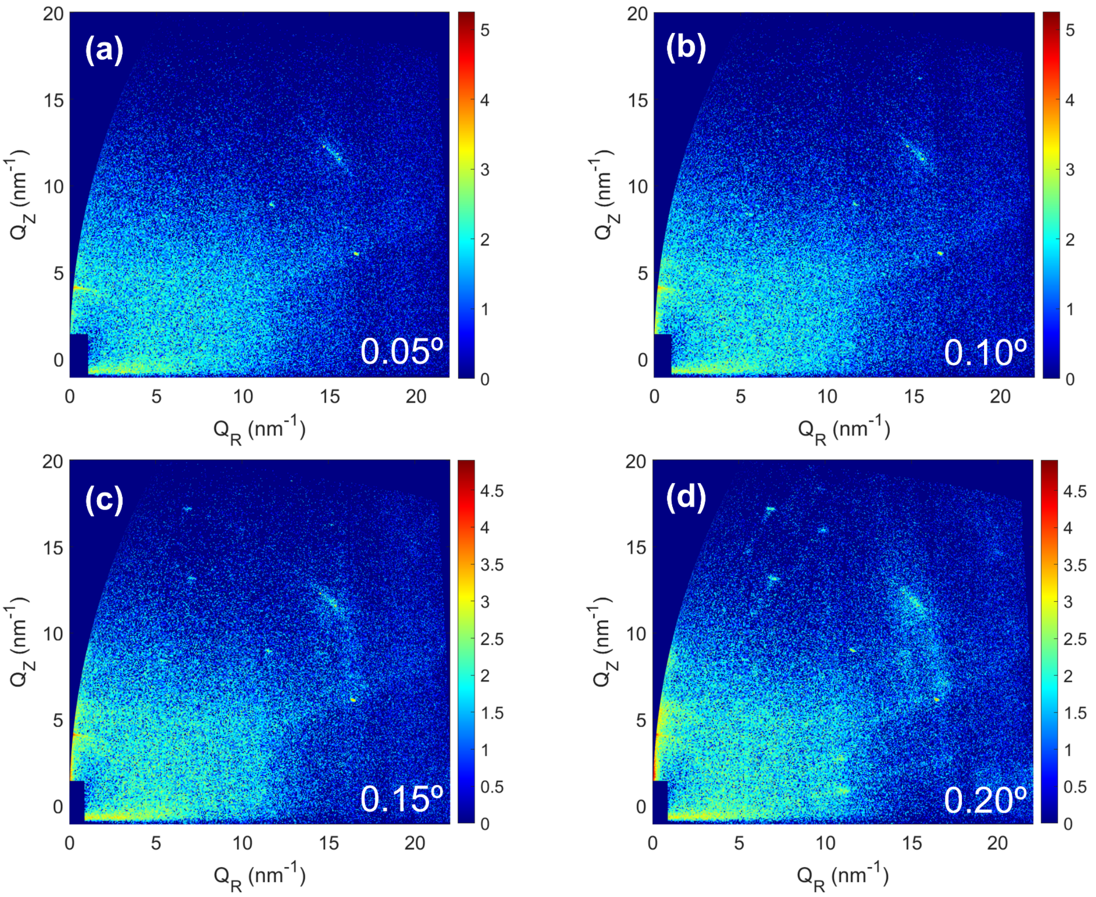

Figure S32. Depth-dependent GIWAXS images recorded for a 2DP $(C5)_2PbI_4$ thin film. The incident angle is indicated in the figures: (A) 0.05°, (B) 0.10°, (C) 0.15°, (D) 0.20°.

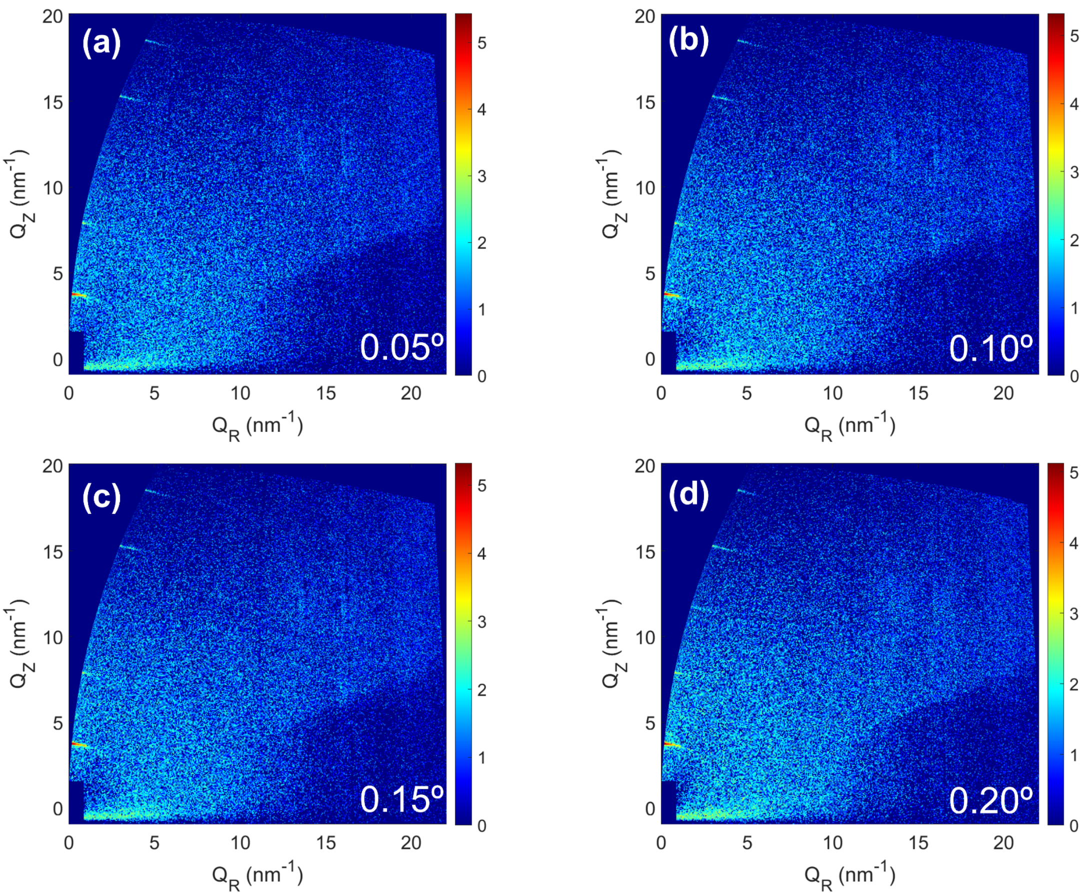

Figure S33. Depth-dependent GIWAXS images recorded for a 2DP $(C6)_2PbI_4$ thin film. The incident angle is indicated in the figures: (A) 0.05°, (B) 0.10°, (C) 0.15°, (D) 0.20°.

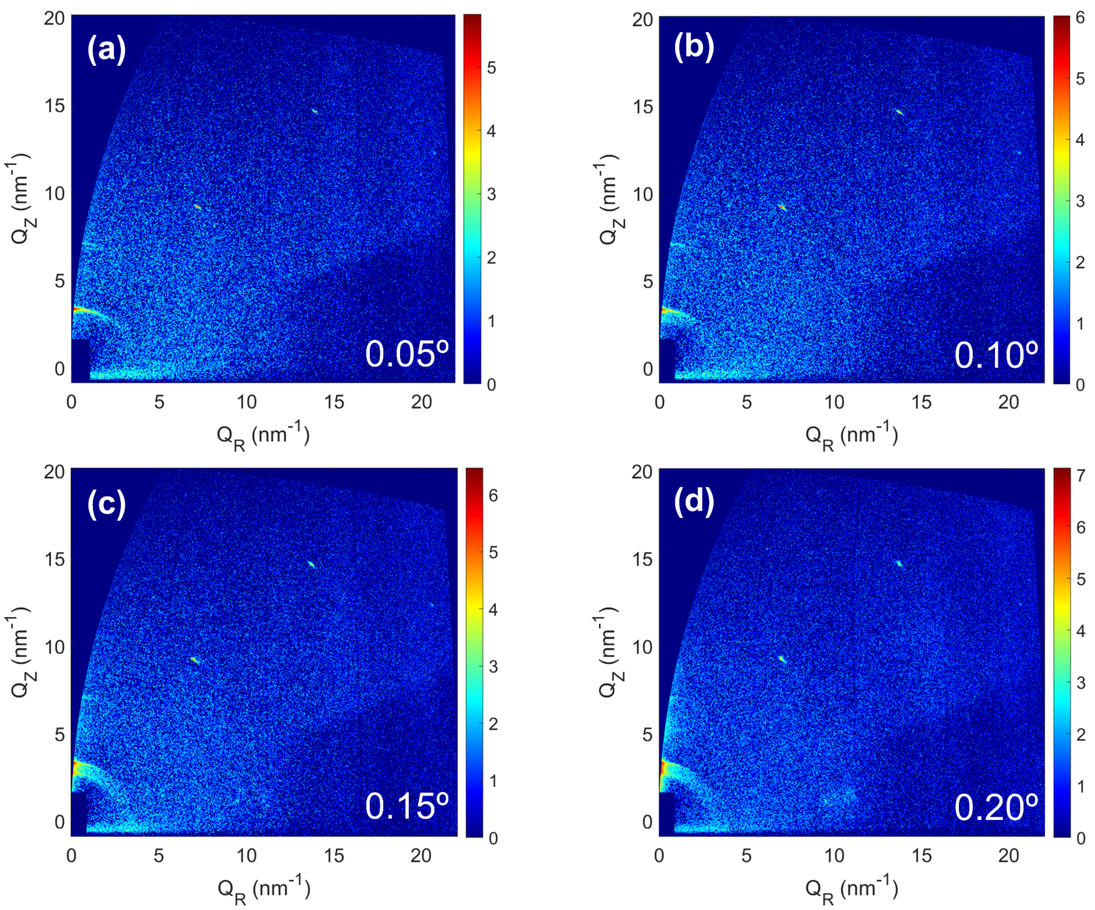

Figure S34. Depth-dependent GIWAXS images recorded for a 2DP $(C7)_2PbI_4$ thin film. The incident angle is indicated in the figures: (A) 0.05°, (B) 0.10°, (C) 0.15°, (D) 0.20°.

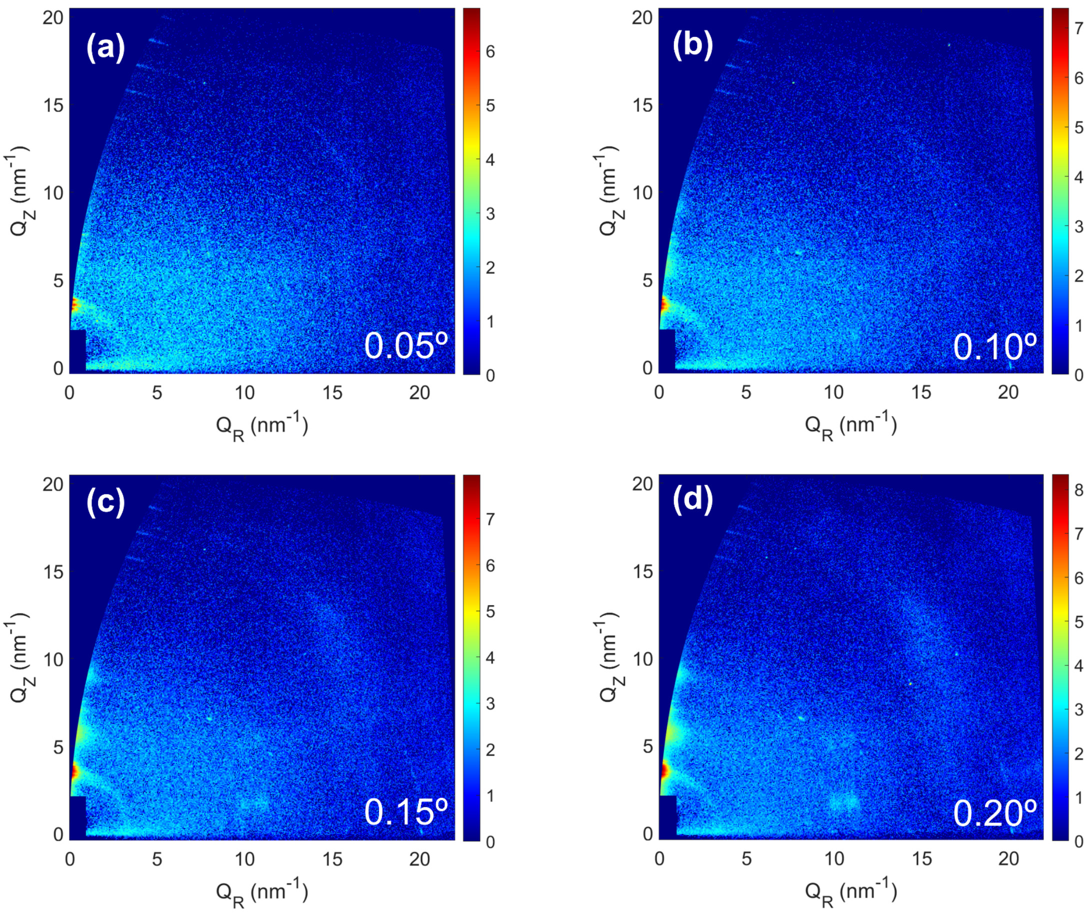

Figure S35. Depth-dependent GIWAXS images recorded for a 2DP $(C8)_2PbI_4$ thin film. The incident angle is indicated in the figures: (A) 0.05°, (B) 0.10°, (C) 0.15°, (D) 0.20°.

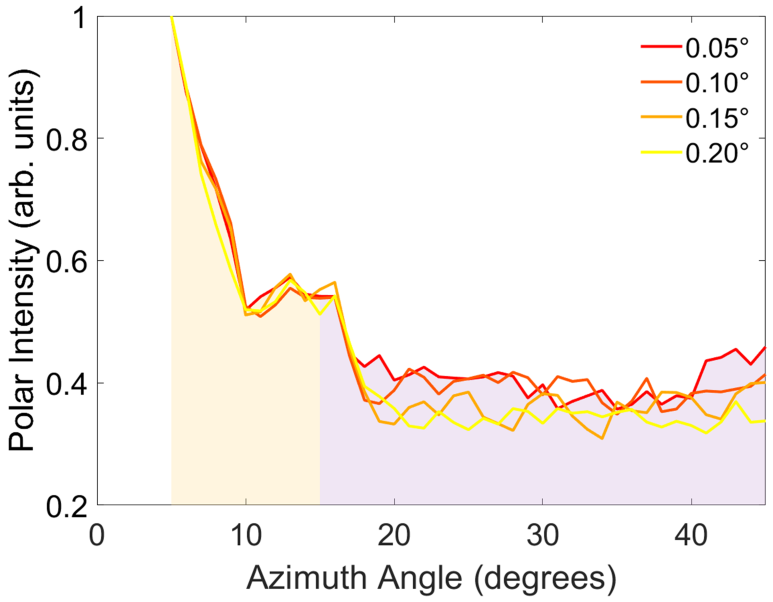

Figure S36. Polar intensity profiles of the (002) diffraction peak for a 2DP $(C3)_2PbI_4$ thin film collected at incident angles ranging from 0.05° (probing the film surface) to 0.20° (penetrating through the entire film).

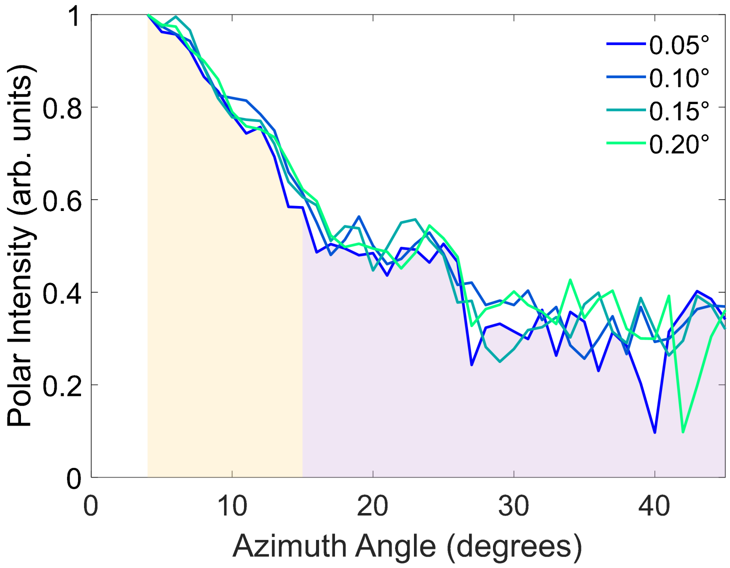

Figure S37. Polar intensity profiles of the (002) diffraction peak for a 2DP $(C6)_2PbI_4$ thin film collected at incident angles ranging from 0.05° (probing the film surface) to 0.20° (penetrating through the entire film).

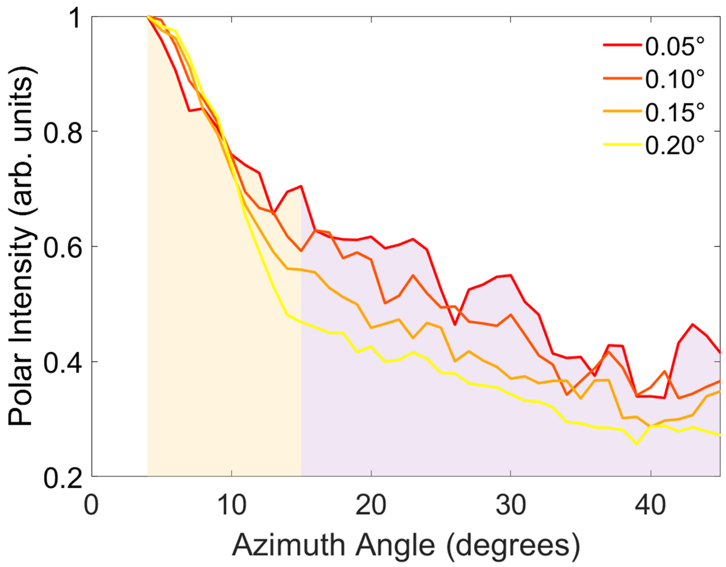

Figure S38. Polar intensity profiles of the (002) diffraction peak for a 2DP $(C7)_2PbI_4$ thin film collected at incident angles ranging from 0.05° (probing the film surface) to 0.20° (penetrating through the entire film).

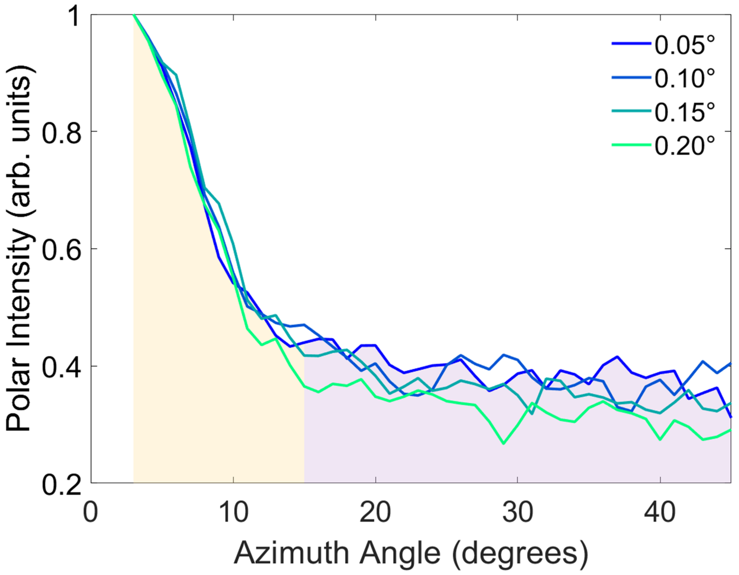

Figure S39. Polar intensity profiles of the (002) diffraction peak for a 2DP $(C8)_2PbI_4$ thin film collected at incident angles ranging from 0.05° (probing the film surface) to 0.20° (penetrating through the entire film).

8.2 Depth-dependent GIWAXS measurements

The X-ray penetration depth is estimated from the following equation[15]:

$$\tau_{(\alpha)} = \frac{\sqrt{2}\lambda}{4\pi}\left\{\sqrt{(\alpha^2 - \alpha_c^2) + 4\beta^2} - (\alpha^2 - \alpha_c^2)\right\}^{-\frac{1}{2}} \quad (11)$$

where λ is the wavelength of the X-rays, α is the incident angle, and $\alpha_c$is the critical angle of the perovskite film. The critical angle $\alpha_c$can be obtained from:

$$\alpha_c = \sqrt{2\delta}$$

where the parameters $\delta$and β are calculated from the atomic scattering factors (f_1 and f_2) as follows:

$$\delta = \frac{n_a r_e \lambda^2}{2\pi} f_1$$

$$\beta = \frac{n_a r_e \lambda^2}{2\pi} f_2$$

Here n_a is the number density and r_e is the classical electron radius of 2.818×10^{-15} m. The X-ray wavelength λ was 1.54 Å for the Cu Kα source used in this study. The atomic scattering factors, physical density of the perovskites, and molar mass were obtained from literature.[14].

The relationship between the X-ray penetration depth and the incident angle for C3–C8 thin films is shown in Figure S40 - S45. Although the critical angle $\alpha_c$varies slightly among different samples, its values remain close (0.09°–0.13°). Therefore, to investigate the variation in layer orientation in our 2D perovskite films, incident angles ranging from 0.05° (surface-sensitive) to 0.20° (probing through the full film thickness) were selected for the GIWAXS measurements.

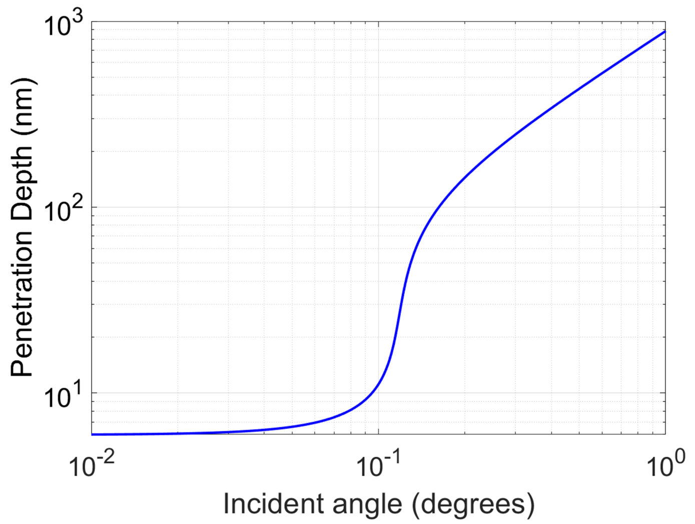

Figure S40. The relationship between X-ray penetration depth and incident angle for 2DP $(C3)_2PbI_4$ thin films.

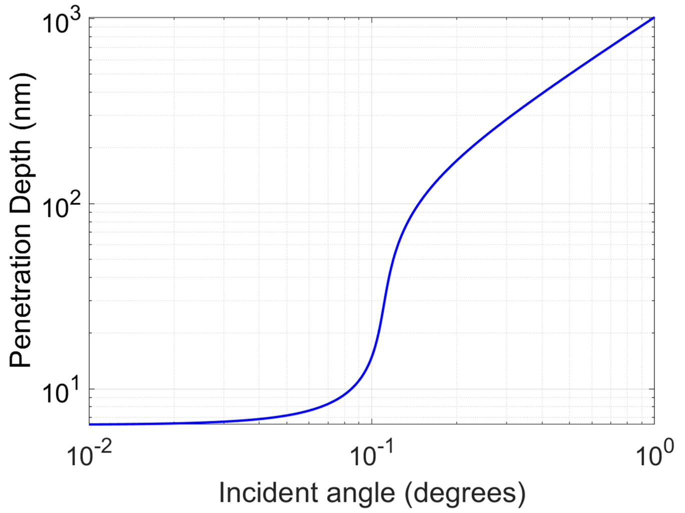

Figure S41. The relationship between X-ray penetration depth and incident angle for 2DP $(C4)_2PbI_4$ thin films.

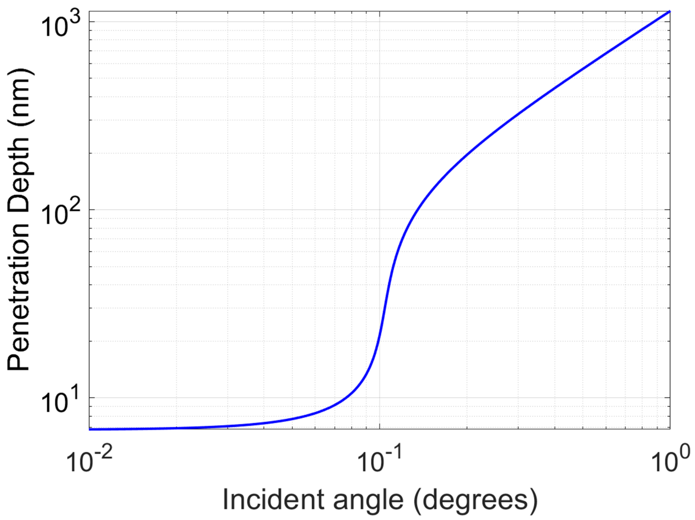

Figure S42. The relationship between X-ray penetration depth and incident angle for 2DP $(C5)_2PbI_4$ thin films.

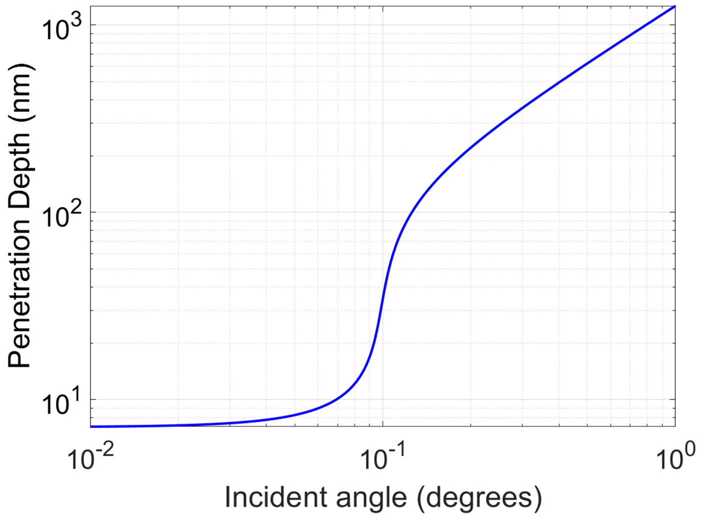

Figure S43. The relationship between X-ray penetration depth and incident angle for 2DP $(C6)_2PbI_4$ thin films.

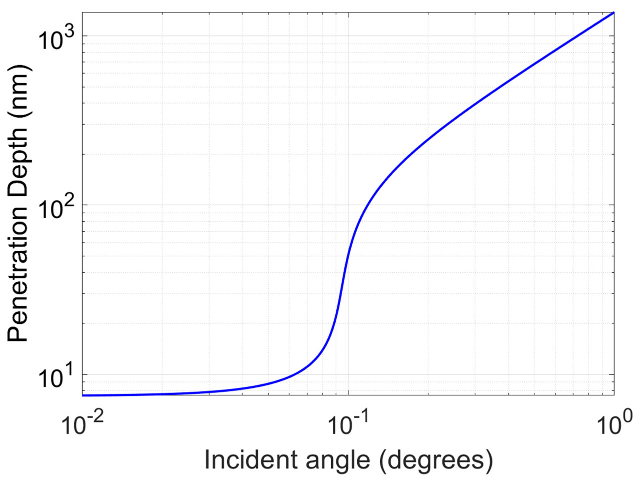

Figure S44. The relationship between X-ray penetration depth and incident angle for 2DP $(C7)_2PbI_4$ thin films.

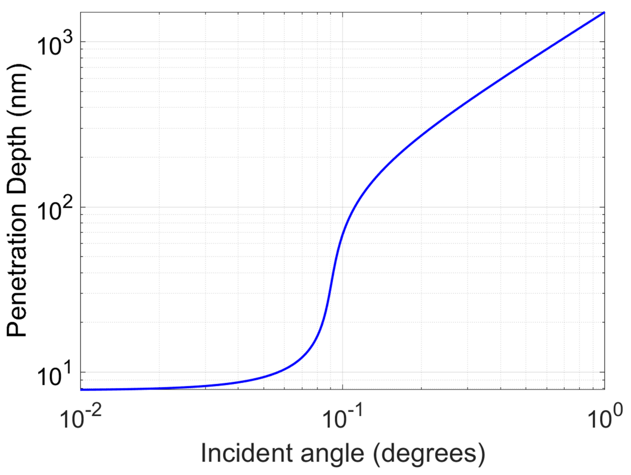

Figure S45. The relationship between X-ray penetration depth and incident angle for 2DP $(C8)_2PbI_4$ thin films.

References

1. Choghaei, M.; Schiffer, M.; Tyagi, V.; Righetto, M.; Du, J.; Buchmüller, M.; Brinkmann, K. O.; Brocks, G.; Görrn, P.; Herz, L. M.; Tao, S.; Riedl, T.; Olthof, S., Odd-even effects in lead-iodide-based Ruddlesden–Popper 2D perovskites. *Journal of Materials Chemistry A* **2025,** *13* (24), 18935-18947.
2. Du, J.; Righetto, M.; Kober-Czerny, M.; Yan, S.; Elmestekawy, K. A.; Snaith, H. J.; Johnston, M. B.; Herz, L. M., Inter-Layer Diffusion of Excitations in 2D Perovskites Revealed by Photoluminescence Reabsorption. *Advanced Functional Materials* **2025,** *35* (26), 2421817.
3. Butler-Caddle, E.; Jayawardena, K. D. G. I.; Wijesekara, A.; Milot, R. L.; Lloyd-Hughes, J., Distinguishing carrier transport and interfacial recombination at perovskite/transport-layer interfaces using ultrafast spectroscopy and numerical simulation. *Physical Review Applied* **2024,** *22* (2), 024013.
4. Yamada, Y.; Yamada, T.; Phuong le, Q.; Maruyama, N.; Nishimura, H.; Wakamiya, A.; Murata, Y.; Kanemitsu, Y., Dynamic Optical Properties of CH(3)NH(3)PbI(3) Single Crystals As Revealed by One- and Two-Photon Excited Photoluminescence Measurements. *Journal of the American Chemical Society* **2015,** *137* (33), 10456-9.
5. Kahmann, S.; Tekelenburg, E. K.; Duim, H.; Kamminga, M. E.; Loi, M. A., Extrinsic nature of the broad photoluminescence in lead iodide-based Ruddlesden-Popper perovskites. *Nat Commun* **2020,** *11* (1), 2344.
6. Wu, X.; Trinh, M. T.; Niesner, D.; Zhu, H.; Norman, Z.; Owen, J. S.; Yaffe, O.; Kudisch, B. J.; Zhu, X. Y., Trap states in lead iodide perovskites. *J Am Chem Soc* **2015,** *137* (5), 2089-96.
7. Seitz, M.; Meléndez, M.; Alcázar-Cano, N.; Congreve, D. N.; Delgado-Buscalioni, R.; Prins, F., Mapping the Trap-State Landscape in 2D Metal-Halide Perovskites Using Transient Photoluminescence Microscopy. *Advanced Optical Materials* **2021,** *9* (18), 2001875.
8. Cho, C.; Feldmann, S.; Yeom, K. M.; Jang, Y. W.; Kahmann, S.; Huang, J. Y.; Yang, T. C.; Khayyat, M. N. T.; Wu, Y. R.; Choi, M.; Noh, J. H.; Stranks, S. D.; Greenham, N. C., Efficient vertical charge transport in polycrystalline halide perovskites revealed by four-dimensional tracking of charge carriers. *Nature Materials* **2022,** *21* (12), 1388-1395.
9. Yuan, Y.; Yan, G.; Akel, S.; Rau, U.; Kirchartz, T., Deriving mobility-lifetime products in halide perovskite films from spectrally and time-resolved photoluminescence. *Science advances* **2025,** *11* (16), eadt1171.
10. Wei, Z.; Bayikadi, K. S.; Mamak, C.; Dubajic, M.; Huang, C.-S.; Pan, L.; Kanatzidis, M. G.; Stranks, S. D., Carrier Diffusion Links Single Crystal Quality and Photoluminescence in Halide Perovskite Radiation Detectors. *Advanced Materials* **2026,** *38* (4), e12302.
11. Seitz, M.; Magdaleno, A. J.; Alcazar-Cano, N.; Melendez, M.; Lubbers, T. J.; Walraven, S. W.; Pakdel, S.; Prada, E.; Delgado-Buscalioni, R.; Prins, F., Exciton diffusion in two-dimensional metal-halide perovskites. *Nat Commun* **2020,** *11* (1), 2035.
12. Wehrenfennig, C.; Liu, M.; Snaith, H. J.; Johnston, M. B.; Herz, L. M., Charge-carrier dynamics in vapour-deposited films of the organolead halide perovskite CH3NH3PbI3−xClx. *Energy & Environmental Science* **2014,** *7* (7), 2269-2275.
13. Qin, M.; Chan, P. F.; Lu, X., A Systematic Review of Metal Halide Perovskite Crystallization

and Film Formation Mechanism Unveiled by In Situ GIWAXS. *Advanced Materials* **2021,** *33* (51), e2105290.
14. Qin, M.; Xue, H.; Zhang, H.; Hu, H.; Liu, K.; Li, Y.; Qin, Z.; Ma, J.; Zhu, H.; Yan, K.; Fang, G.; Li, G.; Jeng, U. S.; Brocks, G.; Tao, S.; Lu, X., Precise Control of Perovskite Crystallization Kinetics via Sequential A-Site Doping. *Advanced Materials* **2020,** *32* (42), e2004630.
15. Steele, J. A.; Solano, E.; Hardy, D.; Dayton, D.; Ladd, D.; White, K.; Chen, P.; Hou, J.; Huang, H.; Saha, R. A.; Wang, L.; Gao, F.; Hofkens, J.; Roeffaers, M. B. J.; Chernyshov, D.; Toney, M. F., How to GIWAXS: Grazing Incidence Wide Angle X-Ray Scattering Applied to Metal Halide Perovskite Thin Films. *Advanced Energy Materials* **2023,** *13* (27), 2300760.
